# Müller Glia Activation in Response to Inherited Retinal Degeneration Is Highly Varied and Disease-Specific

**DOI:** 10.1371/journal.pone.0120415

**Published:** 2015-03-20

**Authors:** Claire Hippert, Anna B. Graca, Amanda C. Barber, Emma L. West, Alexander J. Smith, Robin R. Ali, Rachael A. Pearson

**Affiliations:** 1 Department of Genetics, University College London Institute of Ophthalmology, 11–43 Bath Street, London, EC1V 9EL, United Kingdom; 2 NIHR Biomedical Research Centre at Moorfields Eye Hospital NHS Foundation Trust and UCL Institute of Ophthalmology, City Road, London, EC1V 2PD, United Kingdom; Universidade Federal do Rio de Janeiro, BRAZIL

## Abstract

Despite different aetiologies, most inherited retinal disorders culminate in photoreceptor loss, which induces concomitant changes in the neural retina, one of the most striking being reactive gliosis by Müller cells. It is typically assumed that photoreceptor loss leads to an upregulation of glial fibrilliary acidic protein (Gfap) and other intermediate filament proteins, together with other gliosis-related changes, including loss of integrity of the outer limiting membrane (OLM) and deposition of proteoglycans. However, this is based on a mix of both injury-induced and genetic causes of photoreceptor loss. There are very few longitudinal studies of gliosis in the retina and none comparing these changes across models over time. Here, we present a comprehensive spatiotemporal assessment of features of gliosis in the degenerating murine retina that involves Müller glia. Specifically, we assessed Gfap, vimentin and chondroitin sulphate proteoglycan (CSPG) levels and outer limiting membrane (OLM) integrity over time in four murine models of inherited photoreceptor degeneration that encompass a range of disease severities (*Crb1^rd8/rd8^, Prph2^+/Δ307^, Rho^-/-^, Pde6b^rd1/rd1^*). These features underwent very different changes, depending upon the disease-causing mutation, and that these changes are not correlated with disease severity. Intermediate filament expression did indeed increase with disease progression in *Crb1^rd8/rd8^* and *Prph2^+/Δ307^*, but decreased in the *Prph2^+/Δ307^* and *Pde6b^rd1/rd1^* models. CSPG deposition usually, but not always, followed the trends in intermediate filament expression. The OLM adherens junctions underwent significant remodelling in all models, but with differences in the composition of the resulting junctions; in *Rho^-/-^* mice, the adherens junctions maintained the typical rod-Müller glia interactions, while in the *Pde6b^rd1/rd1^* model they formed predominantly between Müller cells in late stage of degeneration. Together, these results show that gliosis and its associated processes are variable and disease-dependent.

## Introduction

The vast majority of degenerative retinal diseases lead either directly or indirectly to the loss of photoreceptor cells. As degeneration progresses so the microenvironment of the retina undergoes a number of significant changes. The loss of photoreceptors causes the cytoarchitecture of the outer nuclear layer (ONL) to become disrupted and the outer limiting membrane (OLM), a network of adherens junctions formed between photoreceptors and Müller glia, may become compromised. In addition, Müller glial cells undergo reactive gliosis, leading to the formation of a glial scar that can envelope the entire retina at late stages of degeneration [1,2]. This scar can act as a reservoir for the accumulation of extracellular matrix (ECM) proteins including chondroitin sulphate proteoglycans (CSPGs), which are known to be inhibitory to axonal regeneration [3,4]. Each of these processes is likely to have a significant impact upon the retina and its health and physiology. Moreover, having a complete understanding of such changes is essential for the development of promising new therapeutic strategies including gene [5] and cell [6] replacement. Previously, retinal gliosis has been shown to negatively impact on the efficiency of viral transduction in gene therapy [7], the integration of transplanted photoreceptors ([8], reviewed in [9]) and the ability of retinal grafts [10] and electronic implants [11] to contact the underlying retina. Elsewhere in the CNS, reactive gliosis has long been considered as the major impediment to axonal regrowth after an injury [12,13]. Nonetheless, the formation of a glial barrier around a lesion site is also an advantage, because it isolates the still intact CNS tissue from secondary lesions. In addition, there are reports to suggest that in certain conditions reactive astrocytes could even provide a permissive substratum for neurite extension (reviewed in [14]). For these reasons, understanding the process of glial scar formation, how this process differs in different models of degeneration, and finding strategies to circumvent these barriers, represent major challenges to the advancement of many ocular therapies.

Typically, gliosis is characterized by a dramatic increase in intermediate filament expression and a pronounced hypertrophy of Müller cells [15]. In addition to the upregulation of the intermediate filament proteins, glial fibrillary acidic protein (Gfap) and vimentin, reactive Müller cells may undergo hypertrophy, presenting a proliferation of fibrous processes and deposition of proteoglycans, particularly CSPGs, at the outer edge of the retina [16–18]. This process of gliosis is characteristic of many retinal disease models [19–21], although the temporal relationship between the onset of gliosis and degeneration may vary between disease models. To our knowledge, no comparison between models at equivalent stages of degeneration has been made. Given the apparent complexities of the gliotic process, more precise dissections of the links between the onset of glial reactivity and progressive neurodegeneration are needed. Here, we present the first comparative characterization of the changes in the microenvironment of the neural retina in four models of inherited retinal degeneration over time. Specifically, we assess the levels of Gfap, vimentin and CSPGs, together with OLM integrity and ONL architecture, to provide a more comprehensive analysis of the presence and distribution of these proteins in the degenerating retina. Our data show that different initiating genetic defects can lead to striking differences in the response by Müller glia.

## Materials and Methods

### Ethics statement

All animal studies were carried out under the Animals (Scientific Procedures) Act 1986 under a project license PPL 70/8120 issued by the UK Government Home Office and conducted in accordance with protocols approved by the Animal Welfare and Ethics Committee of the UCL Institute of Ophthalmology. All animals were killed by trained personnel using cervical dislocation (approved under Schedule 1 as a method of humane killing). All efforts were made to minimize the number and suffering of animals used in these experiments.

### Animals

C57Bl/6J (Harlan, UK), *Crb1* ^*rd8/rd8*^ (C57Bl/6J background; Jackson Laboratory, USA), *Rho* ^-/-^ (C57Bl/6J background; P. Humphries, Trinity College Dublin, Republic of Ireland), *Pde6b* ^*rd1/rd1*^ (C3H/HeJ background; Harlan, UK), and *Prph2* ^*+/Δ307*^ (C57Bl/6J background; J. Farrar, Trinity College Dublin, Republic of Ireland) mice were maintained in the animal facility at University College London. Animals of both sexes were kept on a standard 12/12 hour light/dark cycle and at the same light levels throughout and used at the ages specified in Table 1.

**Table 1:** Summary of the different models and stages of retinal degeneration studied.

Mouse model	Early Degeneration(ONL >70% of WT)	Mid Degeneration(ONL 30–70% of WT)	Late Degeneration(ONL <30% of WT)	Mouse model description
^$^ *C57Bl/6J* (WT)	6 weeks	6 months	12 months	*Harlan*, *UK*
* *Crb1* ^*rd8/rd8*^	3 weeks	6 weeks	12 weeks	[22]
*Prph2* ^*+/Δ307*^	2 months	4 months	6 months	[23]
*Rho* ^-/-^	4 weeks	6 weeks	10 weeks	[24]
*Pde6b* ^*rd1/rd1*^	10 days	3 weeks	6–8 weeks	[25]

WT, wild-type.

^$^ Wild-type is stationary.

* *Crb1* ^*rd8/rd8*^ undergoes focal degeneration on this background, which broadly correlates with the stages outlined here.

### Immunohistochemistry and Histology

Eyes were dissected out after administering a small burn to the overlying sclera to provide a landmark for the superior retina. The eye cups were then carefully orientated and embedded in a standardized fashion in OCT (TissueTek) before being left over night at -20°C and then cut as transverse sections 18 μm thick. In order to avoid oblique cuts, all images shown are from the central most region of the eye, immediately adjacent to or through the optic nerve. Immunohistochemistry was performed at the same time for all models/time points for any given marker. Specific details for each antibody and protocols used for immunohistochemistry can be found in S1 Table. Briefly, cryosections were air-dried for 15–30 min and washed in phosphate-buffered saline (PBS). Sections were post-fixed in 1% paraformaldehyde (PFA) for 5 min and then pre-blocked for 1 hr at room temperature (RT) in a blocking solution before being incubated with appropriate primary antibody overnight (o/n) at 4°C. After rinsing with PBS, sections were incubated with secondary antibody for 2 hrs at RT, rinsed and counter-stained with Hoechst 33342. Negative controls omitted the primary antibody.

### Western Blot

Gfap, vimentin and CSPG levels were determined using whole neural retinae dissected from 3 mice per time point per model, snap frozen in liquid nitrogen. Retinal tissue was lysed in RIPA buffer with protease inhibitor (Sigma Aldrich, Gillingham, UK) then cell membranes were disrupted using a sonicator with micro-tip (Soniprep 150, MSE London, UK). Specific details for each antibody used for Western Blot can be found in S2 Table. Briefly, equal amounts of protein (20 μg in Laemmli’s loading buffer) were run on a 12% sodium dodecylsulfate—polyacrylamide (SDS-PAGE) gel for Gfap (50 kDa), Vimentin (58 kDa) and the housekeeping protein Histone 2B (H2B) (15 kDa). Due to the diverse nature of CSPGs and their larger sizes (75–400 kDa) samples were resuspended in non-reducing loading buffer [26] and run on a two-layer SDS-PAGE (12% bottom, 4% top). The separated proteins were electrotransferred to PVDF membranes (Millipore, Watford, UK). The membranes were blocked for 1 hr at RT and then incubated overnight at 4°C with primary antibodies. After washing in PBST, the membranes were incubated in secondary antibody for 1 hr at RT. Chemiluminescence detection was performed using a Fujifilm LAS-1000 Luminescence Image Analyser after incubation with enhanced luminescence reagent (ECL plus GE Healthcare UK Ltd. Amersham, UK). Band intensities were quantified using Image J software and normalized to H2B levels.

### Degeneration assessments

ONL thickness (from outer to inner edge of ONL) and ONL density were measured using single section confocal images (Leica TCS SPE, Leica Microsystems, UK) taken at x80 magnification. Images were acquired from 3 standardised regions of the superior retina immediately adjacent to the optic nerve (herein termed superior-posterior) from at least three independent animals for each model per time point. The rate of degeneration was calculated using the ONL thickness measurements: the total loss of ONL thickness between early and late stage degeneration was divided by the number of days over which the degeneration had taken place. The number of rows of photoreceptor nuclei present in the ONL was also determined from the same images, as a second measure of degeneration speed. ONL density was calculated by counting the number of photoreceptor cell nuclei within an image and normalising this to the total area of ONL; ONL density was then expressed as the number of nuclei per 100 μm^2^. Measurements were taken using ImageJ software and analysed in a fully blinded manner.

### Confocal microscopy

Retinal sections were viewed on a confocal microscope (Leica TCS SPE, Leica Microsystems, Milton Keynes, UK). Images show either single confocal sections or merged projection images of an xyz confocal stack through retinal sections, approximately 15 μm thick, as stated. Individual images were acquired using a 2-frame average and all taken at x40 magnification, except for the OLM integrity studies where the images were taken at x80 magnification. Images were taken in both the superior and inferior retina at standardized regions in the anterior margin, equatorial region and posterior retina immediately adjacent to the optic nerve (see schematics in figures). The same laser intensity, gain and offset settings were used across animals for any given marker.

### Semi-thin and Ultra-thin Sections and Electron Microscopy

Mice were sacrificed and the eyes removed after administering a small burn to the overlying sclera to provide a landmark for the superior retina. Eyes were fixed in 3% glutaraldehyde / 1% PFA buffered to pH 7.4 with 0.08 sodium cacodylate-HCl. The cornea and lens were removed and the eye-cups orientated and processed, as previously described [27,28]. Briefly, following a washing step (15 min; 2.5% glutaraldehyde and 0.1 m cacodylate buffered to pH 7.4), the eyes were osmicated for 2.5 hrs in a 1% aqueous solution of osmium tetroxide in the dark, followed by dehydration steps through ascending ethanol series (50–100%, 10 min per step with rotation). After three changes of 100% ethanol, specimens were passed through propylene oxide (3 x 10 min) and left in a 50:50 mixture of propylene oxide and araldite for a minimum of 3 hrs with rotation at RT. Following a single change to fresh araldite (5 hrs with rotation) the specimens were embedded and cured for 48 hrs at 60°C. Semi-thin (0.7 μm) and ultra-thin (0.07 μm) sections were cut using a Leica Ultracut S microtome fitted with an appropriate diamond knife (Diatome histoknife Jumbo or Diatome Ultra-thin respectively). Ultra-thin sections were collected on copper grids (100 mesh, Agar Scientific, UK), contrast-stained with 1% uranyl acetate and lead citrate and analysed using a JEOL 1010 Transmission Electron Microscope (80 kV), fitted with a digital camera for image capture. Semi-thin sections were stained with 1% toluidine blue and evaluated using a Leitz Diaplan microscope fitted with a Leica digital camera DC 500 for image capture.

### H&E staining

For histological assessment, eyes were removed after administering a small burn to the overlying sclera to provide a landmark for the superior retina. Tissue was fixed in buffered formalin overnight at 4°C. Retinal sections were prepared by step-wise dehydration in isoproponal prior to paraffin embedding (Histocentre). Sections (5 μm thick) were cut in a nasal-temporal direction and affixed to glass slides. Staining using standard haematoxylin and eosin protocols was performed.

### Statistics

All means are stated ± standard deviation, unless otherwise stated. N = number of eyes examined, where appropriate (one eye per animal was used for any given method of assessment). Statistical significance was assessed using a one-way ANOVA test with Dunnett’s (comparing against wild-type) or Tukey’s (inter-group comparison) correction applied for multiple comparisons. P values are presented as p < 0.05 = *, p < 0.01 = ** and p < 0.001 = ***.

## Results

### Changes in retinal cytoarchitecture with degeneration

**Fig 1:**
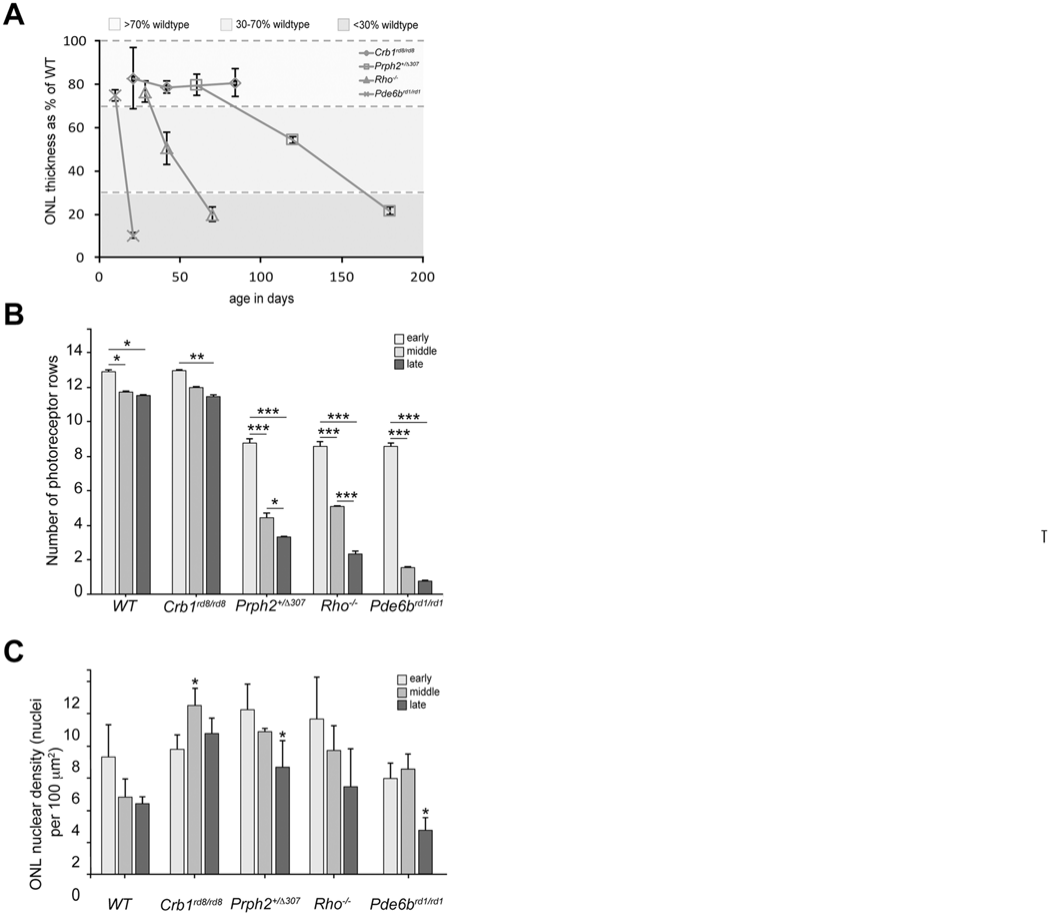
Retinal cytoarchitecture changes with disease progression. Disease severity was assessed in terms of ONL thickness, the rate of cell loss and the resulting photoreceptor cell density. **A**. Photoreceptor loss was the fastest in the *Pde6b* ^*rd1/rd1*^ mouse with an average loss of 3.4 μm per day. The *Rho* ^-/-^ model degenerated at a moderate rate, with an average loss of 0.5 μm per day, while the *Prph2* ^*+/Δ307*^ mouse model underwent a comparatively slow rate of degeneration, with an average loss of 0.06 μm per day. Statistical significance was assessed using a one-way ANOVA test with Dunnett’s correction. **B**. A significant reduction in number of photoreceptor rows was observed in all disease models. In *Crb1* ^*rd8/rd8*^ model, a marked decrease was observed between early and late ages, whereas *Prph2* ^*+/Δ307*^ and *Rho* ^-/-^ showed a significant reduction between each examined stage of degeneration. In *Pde6b* ^*rd1/rd1*^ mice, only a single layer of cones remained at the latest time point examined. Statistical significance was assessed with a one-way ANOVA test with Tukey’s correction. **C**. There was a small but significant reduction in ONL cell density in *Prph2* ^*+/Δ307*^, *Pde6b* ^*rd1/rd1*^ and *Rho* ^-/-^ mice at late, compared to early, stages of degeneration. Conversely, a small but significant increase in cell density was observed in *Crb1* ^*rd8/rd8*^ mice with degeneration. Statistical significance was assessed with a one-way ANOVA test with Tukey’s correction. *P < 0.05, **P < 0.01, and ***P < 0.001.

We assessed gliosis across a number of clinically relevant murine models of inherited retinal disease that represent a range of degeneration rates: three models of retinitis pigmentosa (RP) (*Prph2* ^*+/Δ307*^, *Rho* ^-/-^, *Pde6b* ^*rd1/rd1*^) and a model of Leber Congenital Amaurosis (LCA) (*Crb1* ^*rd8/rd8*^). Each model undergoes a progressive loss of photoreceptors over a period of time (see Table 1). The time points examined were chosen to encompass a range of degeneration stages within each model. These can be described broadly as early degeneration, in which the ONL is >70% of the thickness of the ONL in wild-type retinae, mid-degeneration, where ONL thickness is 30–70% of wild-type and late degeneration, where it is reduced to <30% (Fig. 1A). The *Pde6b* ^*rd1/rd1*^ model undergoes very rapid degeneration and is ~80% of wild-type at postnatal day 10 (P10), a time when neurogenesis is not complete, but is already reduced to 15% by P21. By 6 weeks, the ONL is reduced to a single layer of photoreceptor (cones) nuclei. *Crb1* ^*rd8/rd8*^ on the *C57Bl/6* background undergoes focal degeneration leading to localized photoreceptor loss around the sites of rosette formation. Interestingly, the pseudorosettes that have been reported in the inferior nasal quadrant of the fundus in these mice [19] were also seen in the superior posterior part of the retina. An indication of the rate of degeneration can be obtained by dividing the overall loss of ONL thickness by the number of days between early and late stage (with an exception of using the mid stage for the *Pde6b* ^*rd1/rd1*^ mouse, since there is little change in ONL thickness between mid and late stage). In wild-type mice, ONL thickness is approximately 58 μm (± 7.8) at the early stage examined and stays stable over time. *Pde6b* ^*rd1/rd1*^ mouse has the fastest rate of degeneration of the models examined, with an average loss of 3.4 μm per day. The *Rho* ^-/-^ model degenerates at a moderate rate, with an average loss of 0.5 μm per day, while the *Prph2* ^*+/Δ307*^ and the *Crb1* ^*rd8/rd8*^ mouse models undergo a comparatively slow rate of degeneration, with an average loss of 0.06 μm and 0.016 μm per day, respectively (Fig. 1A). We also assessed disease progression by counting the number of rows of photoreceptor nuclei present over time (Fig. 1B). Similar to the wild-type, *Crb1* ^*rd8/rd8*^ animals undergo a very small but significant reduction in the number of photoreceptor rows over time. Both *Prph2* ^*+/Δ307*^ and *Rho* ^-/-^ mice showed a marked loss between each of the stages examined. As noted above, the *Pde6b* ^*rd1/rd1*^ mouse underwent a very significant loss of photoreceptors between the early and mid time points examined, where the number of rows drops from 9 to about 1.6. Little further loss was observed between the mid and late stages. In addition to reductions in their absolute number, the loss of photoreceptors from the ONL could also affect the density, or packing of the remaining photoreceptors. This may, in turn, affect the cytoarchitecture of the remaining retina. *Prph2* ^*+/Δ307*^ and *Rho* ^-/-^ show a similar loss of density, although this only reached significance in the latest stage examined for *Prph2* ^*+/Δ307*^ mice. A statistically significant reduction in ONL cell density was observed at late, compared to early stage degeneration in *Pde6b* ^*rd1/rd1*^ mice. Conversely, a small but significant increase in cell density with degeneration was observed in *Crb1* ^*rd8/rd8*^ mice (Fig. 1C).

### Changes in intermediate filament levels with degeneration

We first sought to assess changes of the intermediate filament proteins Gfap and vimentin in the different mouse models over time. We used immunohistochemistry (IHC) to perform qualitative assessments of regional changes. In each model, we examined both the superior and inferior retina at the anterior margin, equatorial region and the posterior retina immediately adjacent to the optic nerve (positions indicated in schematics on each figure) at early, mid and late-stage degeneration. Main figures show representative examples of staining at the equatorial region for each model. See Supporting Information S2–S23 Figs. for full representation of all regions examined. Changes in global protein levels were quantified by performing Western Blot (WB) of whole neural retina. H2B was chosen as the loading control since, despite progressive degeneration, levels of this protein remained broadly constant across all time points and models examined, compared to other control markers including Neurofilament 68 (NF-68) and Brain-3b (Brn3b) (S1 Fig.).

**Fig 2:**
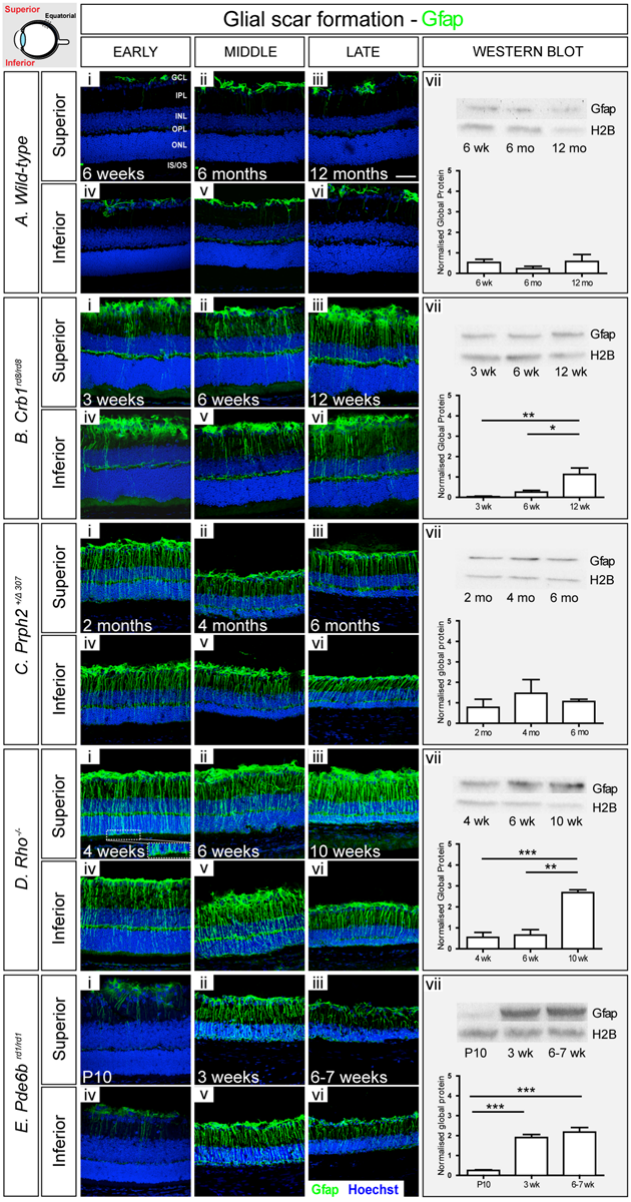
Gfap typically increases with disease progression but decreases in some models in advanced stages of degeneration. **A**. In wild-type retinae, Gfap staining (green) was restricted to astrocytes (i-vi). There were no significant changes in global Gfap levels over time, as determined by Western Blot (vii). **B**. In *Crb1* ^*rd8/rd8*^ model, increasing numbers of Gfap^+ve^ processes were observed with age (i-vi), and this was reflected in a significant increase in global Gfap (vii). **C**. Early stage *Prph2* ^*+/Δ307*^ animals presented with numerous Gfap^+ve^ processes extending into the outer retina, but this was noticeably reduced at late stages (i-vi). A small reduction in a global Gfap levels was also observed (vii). **D**. High levels of Gfap were observed at all examined ages in *Rho* ^-/-^ model with a significant increase between the early and latest stage studied (i-vi). **E**. *Pde6b* ^*rd1/rd1*^ animals showed a bimodal pattern of Gfap synthesis, with staining being particularly strong at the mid stage, but decreasing thereafter (i-vi). A significant increase in global Gfap levels was observed, as determined by Western Blot (vii). Cryosections were immunostained for Gfap (green) and counterstained with nuclei marker Hoechst 33342 (blue). Scale bar, 50 μm. Semiquantitative assessments of Gfap levels in whole neural retina were determined by Western blot. Gfap was normalized against H2B. Statistical significance was assessed with a one-way ANOVA test with Tukey’s correction; *P < 0.05, **P < 0.01, and ***P < 0.001. GCL, ganglion cell layer; IPL, inner plexiform layer; INL, inner nuclear layer; OPL, outer plexiform layer; ONL, outer nuclear layer; IS/OS, photoreceptor inner/outer segment region. A vii, B vii, D vii were first published in *(Barber et al*. *PNAS 2013)*.

**Fig 3:**
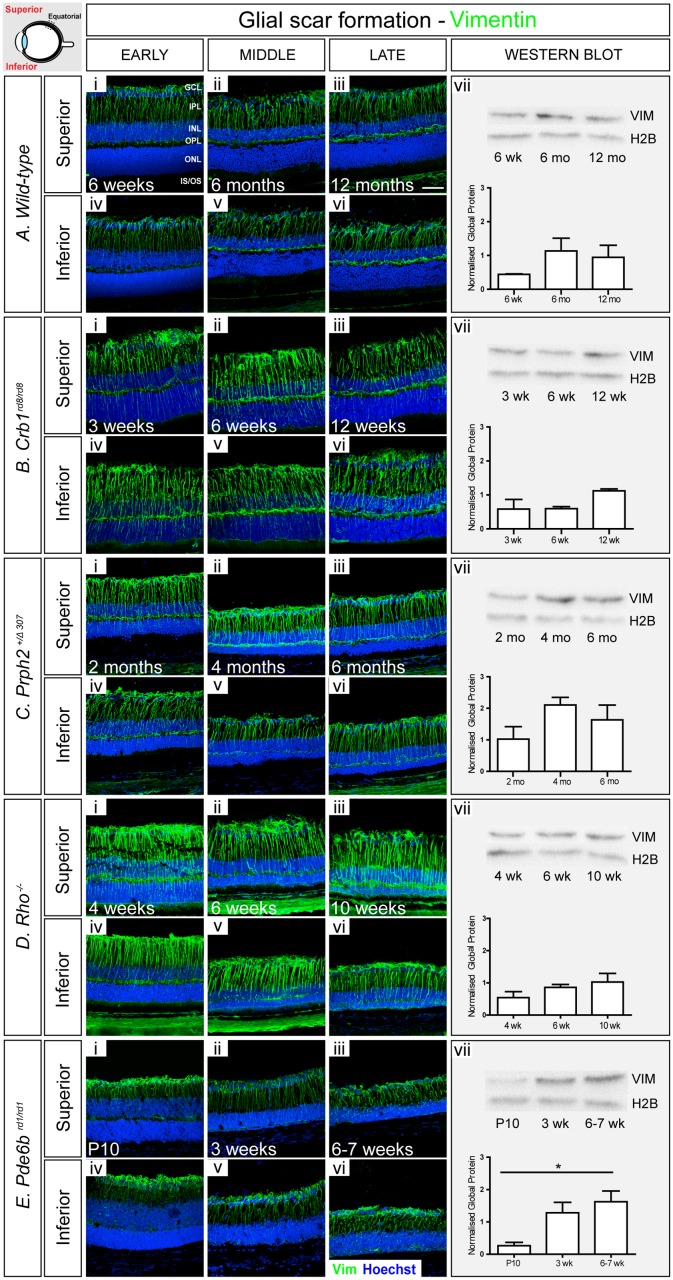
Vimentin increases with disease progression. **A**. In wild-type retinae, vimentin^+ve^ fibers extended throughout the retina at all examined ages (i-vi). Staining at the level of whole retina increased slightly with age (vii). **B**. In *Crb1* ^*rd8/rd8*^ model, the amount of vimentin was higher, particularly in the outer retina, compared to wild-type (i-vi). A small increase in global level was observed at the latest stage examined (vii). **C**. *Prph2* ^*+/Δ307*^ animals showed a similar pattern of vimentin to wild-type, with additional glial processes extending throughout the ONL at the early stage (i-vi), although no significant increase was observed, as determined by Western Blot (vii). **D**. In *Rho* ^-/-^ mice, vimentin^+ve^ processes were numerous and more prominent than in wild-type retinae (i-vi), but no significant change in global level was observed (vii). **E**. In *Pde6b* ^*rd1/rd1*^, robust staining for vimentin was seen throughout (i-vi) and global levels increased significantly with degeneration (vii). Cryosections were immunostained for vimentin (green) and counterstained with nuclei marker Hoechst 33342 (blue). Scale bar, 50 μm. Semiquantitative assessments of vimentin in whole neural retinae were determined by Western blot. Vimentin was normalized against H2B. Statistical significance was assessed with a one-way ANOVA test with Tukey’s correction; *P < 0.05, **P < 0.01, and ***P < 0.001. GCL, ganglion cell layer; IPL, inner plexiform layer; INL, inner nuclear layer; OPL, outer plexiform layer; ONL, outer nuclear layer; IS/OS, photoreceptor inner/outer segment region.

We used *C57Bl/6J* wild-type mice as background-matched (either wholly or partially) non-degenerating controls. As expected, there was little evidence of Gfap presence in either the inferior or superior wild-type retina at 6 weeks of age and this did not change significantly with time (Fig. 2A i-vi; S2 Fig.). Gfap^+ve^ intermediate filaments within the retina were largely restricted to astrocytes at the inner retinal margin, adjacent to the ganglion cell layer (GCL). Some Gfap^+ve^ basal processes of Müller glia were also observed; these were mostly restricted within the anterior margin of the retina and there were no appreciable differences between the inferior and superior retina. WB analysis revealed no significant change in global Gfap across the three ages examined (Fig. 2A vii). In contrast, robust levels of vimentin were observed in both the superior and inferior retina (Fig. 3A i-vi; S3 Fig.). Vimentin^+ve^ Müller glial fibres could be seen extending up into the inner nuclear layer (INL) as far as the outer plexiform layer (OPL) at all ages examined. Interestingly, this appeared to increase with age. Similarly, WB analysis also indicated a trend of increasing global levels of vimentin over time, although this was not statistically significant (Fig. 3A vii).

Degeneration occurs in a focal manner in the *Crb1* ^*rd8/rd8*^ mouse [19]. Similar to the wild-type mouse, only very few Gfap^+ve^ processes were observed in the retina at early and mid-stage disease with the majority restricted to the anterior margins of the retina (Fig. 2B i, ii, iv, v). However, at the latest stage examined, many more Müller glial fibres contained Gfap and the protein extended into the apical processes within the ONL (Fig. 2B iii, vi). Such patterns of Gfap localisation were typical around sites of rosette formation, which were most numerous in the posterior retina (S6 Fig.; arrows). These changes were mirrored by a statistically significant increase in global Gfap (Fig. 2B vii). In contrast, significant levels of vimentin were observed throughout the retina at the earliest stages examined and this was maintained as degeneration progressed (Fig. 3B i-vi; S7 Fig.). A qualitative assessment of vimentin staining indicates that the level was higher in *Crb1* ^*rd8/rd8*^ retinae than in wild-type retinae at all stages examined. Analysis of global vimentin by WB also revealed a slight increase in protein levels at the latest time point examined, although this was not statistically significant (Fig. 3B vii).

When assessing the microenvironment of *Prph2* ^*+/Δ307*^ mice using IHC, we observed markedly higher levels of Gfap at all time points examined, compared to wild-type (Fig. 2C i-vi). Even at the earliest stage examined (2 months), when the ONL is of similar thickness to wild-type, Gfap^+ve^ processes were observed extending throughout the retina to the outer edges of the ONL. Strikingly, however, although there was no significant decrease in global Gfap levels with degeneration as assessed by WB (Fig. 2C vii), IHC revealed that while the basal processes of the Müller glia continue to stain for Gfap, there was a consistent and marked reduction of Gfap in the apical processes over time (Fig. 2C i-vi; S10 Fig.). IHC analysis of vimentin showed a pattern similar to wild-type with time, although overall levels were qualitatively higher at all stages examined, particularly in the basal processes (Fig. 3C i-vi; S11 Fig.). WB revealed a slight increase in global vimentin levels at the latest stages examined compared to early stage of degeneration (Fig. 3C vii).

The *Rho* ^-/-^ mouse presented the most marked upregulation in intermediate filaments of all the models examined. IHC for Gfap showed a significant increase in protein levels as degeneration progressed. Even at the earliest stage examined, the vast majority of Müller glia contained Gfap throughout both basal and apical processes. Moreover, Gfap^+ve^ apical processes were observed extending to the outer edges of the retina and there was evidence of fibre hypertrophy, with processes extending along the outer edge of the retina (Fig. 2D i, magnified area). This pattern of Gfap localisation was seen at all stages examined (Fig. 2D ii, iii, v, vi; S14 Fig.). Interestingly, both basal and apical Müller glial processes appeared thicker compared to other models although further histological analysis would be required to confirm whether this reflects a true morphological change or simply reflects the high levels of Gfap within any given process. WB revealed a statistically significant increase in global Gfap levels between early and late stage disease (Fig. 2D vii). Vimentin presence also increased with age and degeneration, although staining of the apical processes was not as strong as that of Gfap, at least in the earlier time points. As for Gfap, vimentin^+ve^ processes appeared thicker compared to wild-type (Fig. 3D i-vi; S15 Fig.). A trend of global vimentin levels increasing with disease progression was noted, although this was not statistically significant (Fig. 3D vii).

In the *Pde6b* ^*rd1/rd1*^ mouse, IHC indicated a bimodal pattern for *Gfap*, with level being particularly strong at the middle stage, but decreasing thereafter. At the earliest time point examined, P10, the retina is still undergoing neurogenesis. Gfap staining was similar to that seen in adult wild-type retinae and was restricted to a few basal processes only (Fig. 2E i, iv). However, from as early as P15 (S23A–S23F Fig.) onwards the vast majority of Müller cells contained Gfap and protein localisation extended throughout the thickness of the retina into the apical processes (Fig. 2E ii, v). Notably, by very late stage degeneration, when the ONL is reduced to a single layer of cone photoreceptors (6 weeks), Gfap was much reduced, particularly around the ONL (Fig. 2E iii, vi; S18 Fig.). WB analysis also revealed a statistically significant upregulation of global Gfap levels between early and mid-degeneration although the decrease seen by IHC in very late degeneration was not reflected at the global level (Fig. 2E vii). In contrast to the other models of degeneration, vimentin levels appeared to remain fairly low in the *Pde6b* ^*rd1/rd1*^ mouse throughout degeneration and were more similar to wild-type, even at the latest stages examined. Vimentin^+ve^ processes were largely restricted to the inner retina with only few processes extending into the remaining ONL (Fig. 3E i-vi; S19 Fig.). Nonetheless, a statistically significant increase in global vimentin levels was observed between the early and latest time points examined.

### Changes in CSPG levels

**Fig 4:**
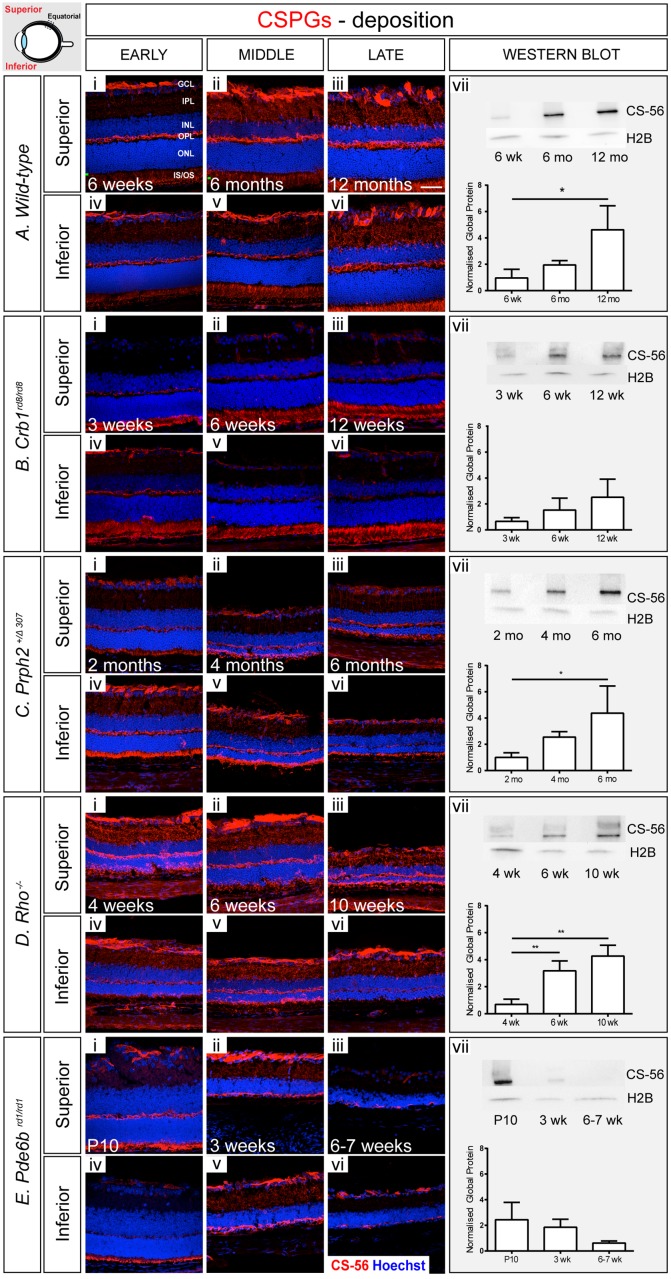
CSPG deposition usually, but not always, follows the trends in Gfap levels. **A**. In a wild-type retina, CSPGs appeared sparsely distributed throughout the segment region and the IPL, and more densely in the OPL and GCL at all time points examined (i-vi). There was a significant change in total CSPG protein levels between early and late ages (vii). **B**. In *Crb1* ^*rd8/rd8*^, CSPG deposition was similar to that observed in wild-type (i-vii), with a small but non-significant increase with degeneration (vii). **C**. *Prph2* ^*+/Δ307*^ animals showed a similar distribution of CSPGs to wild-type (i-vii), with a significant increase in global protein level with degeneration (vii). **D**. In *Rho* ^-/-^ mice, CSPG staining was more dense, compared to non-degenerative animals (i-vi), with a marked increase in global levels with advancing degeneration (vii). **E**. Notably, CSPG levels decreased in *Pde6b* ^*rd1/rd1*^ model with time (i-vii) despite a significant increase in glial scarring. Cryosections were stained with CS-56 antibody (red) and nuclei marker Hoechst 33342 (blue). Scale bar, 50 μm. Semiquantitative assessments of CSPG in whole neural retinae were determined by Western blot. CSPG level was normalized against H2B. Statistical significance was assessed with a one-way ANOVA test with Tukey’s correction; *P < 0.05, **P < 0.01, and ***P < 0.001. GCL, ganglion cell layer; IPL, inner plexiform layer; INL, inner nuclear layer; OPL, outer plexiform layer; ONL, outer nuclear layer; IS/OS, photoreceptor inner/outer segment region.

We next sought to examine changes in the deposition of CSPGs in the degenerating retina. The CS-56 antibody was used for both IHC and WB. This is a well-characterised antibody, which targets a large panel of CSPGs [3,29,30], providing an overview of CSPG deposition. In wild-type retinae, CSPG was sparsely distributed throughout the photoreceptor inner/outer segment (IS/OS) region and inner plexiform layer (IPL), with much higher levels, exhibited as dense staining, in the outer plexiform layer (OPL) and in the GCL at all time points examined (Fig. 4A i-vi; S4 Fig.). No consistent differences were observed in the pattern of CSPG level between the superior and inferior retina. A gradual increase in CSPGs was observed with age and was particularly noticeable within the IS/OS region. This was mirrored by a statistically significant increase in global CSPG levels with age, as assessed by WB (Fig. 4A vii).

A qualitative assessment of IHC in the *Crb1* ^*rd8/rd8*^ mouse indicated that CSPG deposition, as assessed by CS-56 staining, appeared reduced compared to that seen in the wild-type retina. Protein was most noticeable at the outer edge of the ONL in the IS/OS region (Fig. 4B i-vi), and around sites of presumptive rosette formation (see posterior retina in S8 Fig.; arrows). Notably, and in contrast to the wild-type retina, staining for CSPG in the GCL was largely restricted to the posterior retina with very little signal in equatorial and anterior regions of the retina (S8 Fig.). There were no noticeable differences in the CSPG levels between superior and inferior regions of the retina. WB analysis revealed a trend for increasing global CSPG with age, which correlates with the increase in Gfap and vimentin (Fig. 2B, 3B), although this was not statistically significant.

In *Prph2* ^*+/Δ307*^ mice, the distribution of CSPGs was broadly similar to that seen for wild-type mice and did not change significantly with degeneration. CS-56 staining was evident in the IS/OS region, the OPL, the IPL and the GCL (Fig. 4C i-vi) although appeared to be more intense in the IS/OS region, compared to wild-type, and formed a thin band abutting the ONL indicating a more dense deposition of CSPGs. Interestingly, qualitative assessments of IHC indicated some regional differences between the superior and the inferior retina (S12 Fig.); CSPG levels appeared to be higher within the inferior, compared to the superior retina. When examining global amounts of CSPGs by WB, the *Prph2* ^*+/Δ307*^ animals showed a significant upregulation of CSPGs with disease progression between early and late time-points (Fig. 4C vii).

When assessing the microenvironment of *Rho* ^-/-^ mice by IHC, we found that CSPGs were distributed in a pattern similar to that seen in wild-type eyes at all-time points examined (Fig. 4D i-vi). Staining was particularly noticeable in the IS/OS region and extended into the ONL in the later time points (S16M–S16R Fig.). Surprisingly, CSPG levels were consistently reduced in the inferior part of the retina in the late time point, particularly around the equatorial region (Fig. 4D vi; S16Q Fig.). Interestingly, for each stage studied, CSPG staining seemed stronger in the GCL, compared to the same region in the wild-type. WB analysis revealed a significant increase in global CSPGs levels between early and mid/late degeneration (Fig. 4D vii).

In the *Pde6b* ^*rd1/rd1*^ mice, the distribution of CS-56 staining was initially quite similar to wild-type, despite the earliest stage examined coinciding with a period of late developmental changes (P10). However, the intensity of staining was stronger in the IS/OS region compared to wild-type and formed a thin band at the outer margin of the ONL (Fig. 4E i, iv; S20A–S20F Fig.). By mid-stage degeneration, CSPGs were seen throughout the remaining ONL, extending into the OPL, before decreasing at very late stages of degeneration (Fig. 4E ii, iii, v, vi; S20G–S20R Fig.). Notably, CSPG deposition was also significantly reduced in the inner retina, with little CS-56 staining in the astrocytes at the inner retinal margin. These changes were mirrored by a reduction in global CSPG levels over time (Fig. 4E vii).

### Changes in OLM integrity

**Fig 5:**
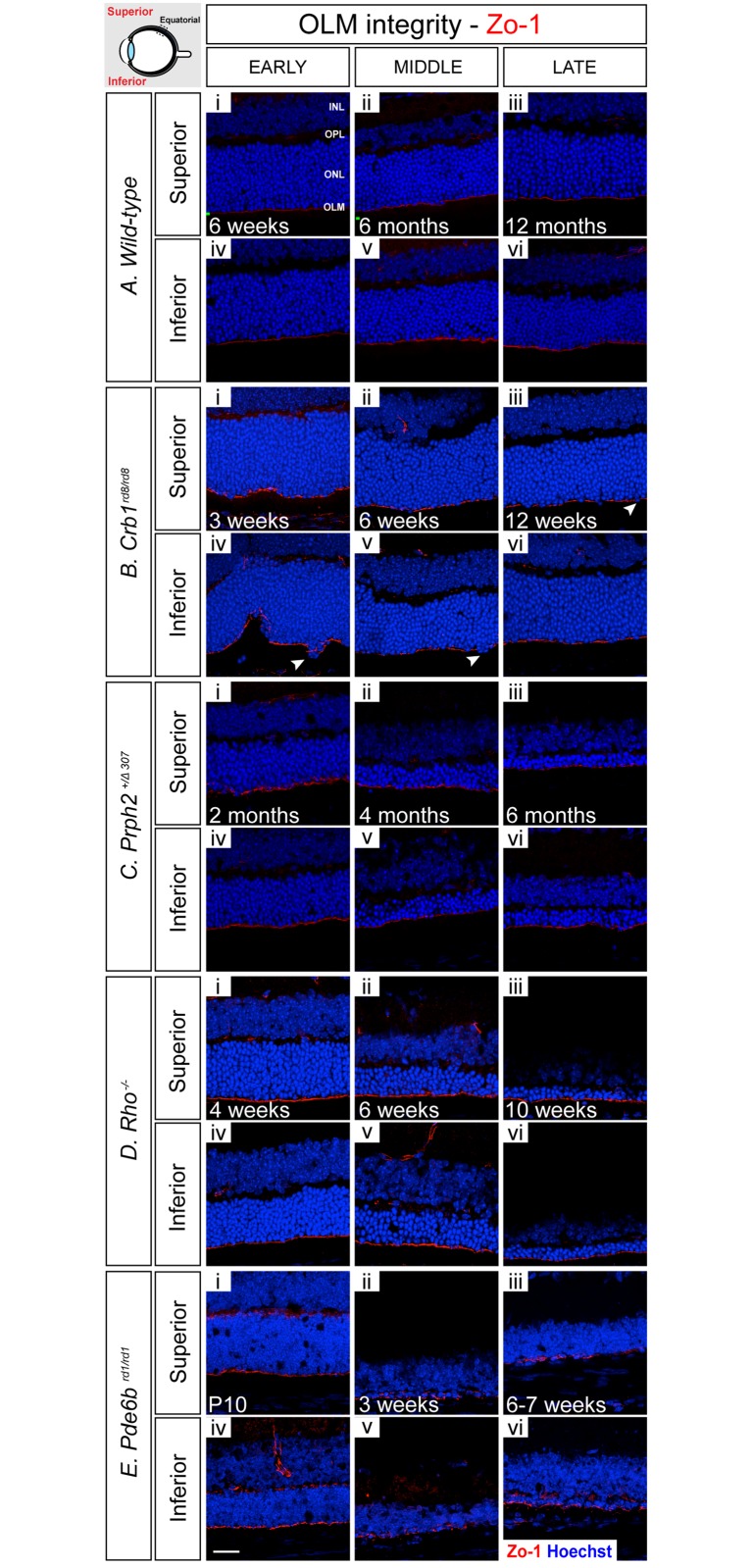
OLM integrity, as assessed by adherens junction adaptor protein staining, is largely maintained during retinal degeneration. **A** i-vi). In wild-type retinae, Zo-1 staining appeared as a continuous line, indicating that the OLM is intact, and remained so at all ages examined. **B**. i-vi) In *Crb1* ^*rd8/rd8*^ animals, Zo-1 staining was noticeably fragmented at all time points examined, indicating significant OLM disruption. **C**. i-vi) In *Prph2* ^*+/Δ307*^ animals model, the OLM was disrupted as indicated by fragmented Zo-1 staining. **D**. (i-vii) In *Rho* ^-/-^ mice, no major changes within the OLM were observed at early and mid-stage of degeneration, but some fragmentation developed by late stage of degeneration. **E**. (i-vi) In *Pde6b* ^*rd1/rd1*^ mice, staining for Zo-1 presented as a strong and continuous line at the earliest time point examined, P10, but became increasingly uneven with degeneration. Cryosections were immunostained for Zo-1 (red) and counterstained with nuclei marker Hoechst 33342 (blue). Scale bar, 25 μm. INL, inner nuclear layer; OPL, outer plexiform layer; ONL, outer nuclear layer; OLM, outer limiting membrane.

**Fig 6:**
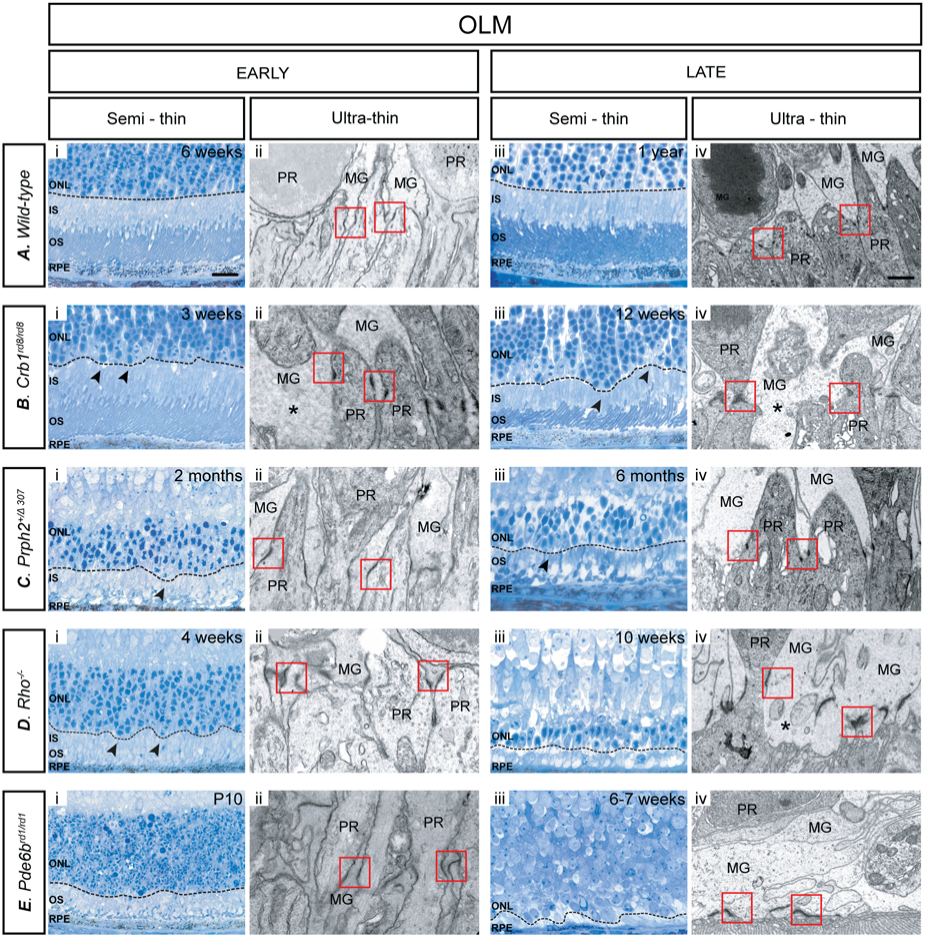
OLM adherens junctions undergo significant remodelling during retinal degeneration in order to maintain OLM integrity. **A**. In wild-type retinae, the OLM presented as a neat continuous line at the outer edge of the ONL, which remained unbroken throughout all the stages examined (i, iii). The majority of adherens junctions were formed between photoreceptor inner segments and Müller glia end feet (ii, iv). **B**. In the *Crb1* ^*rd8/rd8*^ model, the OLM was discontinuous with photoreceptor cell bodies mislocalized in the segment region and/or subretinal space (arrows) (i, iii). Large regions of the outer margin of the ONL were devoid of adherens junctions (asterisks). **C**. In the *Prph2* ^*+/Δ307*^ retina, there was moderate OLM disorganization and mislocalization of photoreceptor cell bodies at both early and late time points (i, iii). While junctions were aligned early on, there was reduction in number and organization of the junctions by the latest age examined (ii, iv). **D**. In early degeneration in the *Rho* ^-/-^ model, there was some minor disorganization of the ONL, with occasional mislocalization of photoreceptor cell bodies (i, iii). Larger gaps between junctions were seen at late, compared with early, degeneration (ii, iv). **E**. In the *Pde6b* ^*rd1/rd1*^ retina, both the ONL and the OLM became markedly disorganized (i, iii). Adherens junction alignment at the outer margin of the ONL was relatively normal at the earliest stage examined but was very disorganized by the latest stage (ii, iv). OLM integrity was assessed at early and late stage of retinal degeneration using semi-thin sections (100x; Scale bar, 25 μm.) and ultrathin electron microscopy (magnification 10,000x; Scale bar, 1 μm). Red boxes: adherens junctions; PR—photoreceptor; MG—Müller glia; arrows indicate displaced photoreceptors; ONL continuity—black dashed line marked on semi—thin sections.

OLM integrity was examined by performing IHC for Zo-1 (Fig. 5) and Crb1 (data not shown), both components of the adherens junctions that forming the OLM [31,32]. This was further confirmed by ultrastructural analysis of electron micrographs and images taken from semi-thin and ultra-thin sections of the earliest and latest time points examined for each model (Fig. 6). In the wild-type inferior and superior retina, at 6 weeks of age (early) Zo-1 staining appears as a continuous line, indicating that the OLM is intact, and remained so at all time points examined (Fig. 5A i-vi; S5 Fig.). Crb1 followed the same staining pattern of Zo-1 in all models (data not shown), with the obvious exception of *Crb1* ^*rd8/rd8*^ animals. Analysis of semi-thin sections demonstrated that, in wild-type retinae, the OLM forms a neat continuous line at the outer edge of the ONL, which remains unbroken throughout the time frame examined (Fig. 6A i, iii, dashed black line). Assessment at the EM level confirms previous reports that the vast majority of these adherens junctions are formed between photoreceptor inner segments and Müller glia end feet, creating a neatly aligned OLM structure (Fig. 6A ii, iv, dashed red boxes).

In both the inferior and superior retina of the *Crb1* ^*rd8/rd8*^ mouse, the staining of Zo-1 appeared fragmented at all time points examined, indicating that the integrity of the OLM is compromised throughout. Crb1 staining was, as expected for this model (Mehalow et al., 2008), absent or diffuse (data not shown). Some photoreceptor cell bodies were also displaced into the subretinal space (Fig. 5B i-vi, arrows; S9 Fig.), an observation also made in semi-thin sections. In the latter, the OLM did not present as a straight line, as in the wild-type, again supporting a loss of OLM integrity and altered layering of photoreceptor nuclei (Fig. 6B i, iii, arrows). Upon closer inspection at the ultrastructural level, some adherens junctions between Müller cells and photoreceptor cells remained (Fig. 6B ii, iv, dashed red boxes) but large regions of the outer margin of the ONL were devoid of adherens junctions (Fig. 6B iv. asterisks).

In the *Prph2* ^*+/Δ307*^ retina, the OLM in both the inferior and superior retina appeared disrupted at all time points examined, as indicated by marked disruptions in Zo-1 staining (Fig. 5C i-vi; S13 Fig.). Semi-thin sections also indicated that there is moderate disorganization at the margin of the ONL including the mislocalization of photoreceptor cell bodies to the IS/OS region at both early and late time points (Fig. 6C i, iii, arrow). Ultrastructural analysis confirmed that while junctions remained quite neatly aligned at both stages examined, they were less numerous in the late stage (Fig. 6C ii, iv). Additional morphological changes in the Müller cells were observed at the late stages of degeneration, including hypertrophy of the end feet.

In contrast to the other models of degeneration examined, the OLM was robustly maintained throughout early and mid-stage of degeneration in the *Rho* ^-/-^ retina. IHC for Zo-1 staining showed a single continuous line at the level of the OLM, which only showed evidence of disruption at the latest stage of degeneration (Fig. 5D i-vi; S17 Fig.). Examination of semi-thin sections shows that there is some minor disorganization in the ONL at early stages of degeneration, compared to wild-type, with some photoreceptor cell bodies being displaced in the subretinal space (Fig. 6D i, iii, arrows). Nonetheless, analysis of electron micrographs shows that despite this disorganization, numerous adherens junctions are clearly visible at the early time point, although there are much larger gaps between junctions by the late time point (Fig. 6D vi, asterisk). By the latest stage examined, when the majority of the photoreceptors have died, much of the space at the outer retinal margin appears to be occupied by the terminal processes of the Müller glia. This is reflected in the nature of the adherens junctions; at the ultrastructural level, many more Müller-to-Müller cell adherens junctions were observed in the late stage, in comparison with early stage degeneration.

In the *Pde6b* ^*rd1/rd1*^ mouse, staining for Zo-1 showed that the OLM is already compromised at the earliest time point examined, P10, a stage where the retina is still maturing, and was markedly fragmented by mid and late stage disease, indicating a significant disruption of the OLM in this model (Fig. 5E i-vi; S21 Fig.). Interestingly, we also observed ectopic presence of Zo-1 at the level of the OPL, between the INL and the remaining ONL from 3 weeks onwards. As might be expected, semi-thin sections showed a significant disorganization within both the ONL and the OLM by the later stages of degeneration (Fig. 6E iii). Analysis at the ultrastructural level (Fig. 6E ii, iv) revealed that the alignment of the adherens junctions at the outer margin of the ONL was relatively normal at the earliest stage examined but was lost by the latest stage. At P10, most junctions were still formed between Müller glia end feet and nascent photoreceptor segments. However, these changes with degeneration and, unsurprisingly in the absence of photoreceptors, by the later stages of degeneration the vast majority of junctions formed are between two Müller glial cells or Müller glial and other retinal cells that could not be confidently identified on the basis of location or morphology due to the extensive degeneration. Together, these results indicate that the OLM undergoes significant re-modelling in this model in the face of almost complete loss of the photoreceptor layer.

## Discussion

The present study presents a comprehensive characterisation of key features of two major retinal modifications that occur during degeneration, namely reactive gliosis and changes in OLM integrity. By examining four models of inherited retinal degeneration at different stages of the degenerative process, we are able to present a spatiotemporal atlas, which illustrates these changes from the onset of disease through to its advanced stages. Our data show that degeneration due to different underlying genetic causes can lead to striking differences in the changes occurring in the retinal microenvironment and the nature of the Müller glial response. Moreover, they show that the magnitude of the glial response is not correlated with disease severity. These observations emphasize that the aetiology of retinal degeneration, including age, retinal cytoarchitecture and disease stage, is likely to represent an important factor that must be considered when developing any clinical intervention.

Gliosis in the retina can be induced by retinal degeneration [10,26], mechanical insult [33], inflammation [34] and/or ageing [35]. Reactive gliosis encompasses a wide range of molecular, biochemical, and morphologic events and is easily demonstrated by IHC due to the overproduction of the intermediate filaments proteins, Gfap and vimentin, and hypertrophy of the terminal processes of Müller glial cells. The increased presence of intermediate filaments is thought to help stabilize the newly formed terminal processes of Müller cells and provide resistance to mechanical stress [36,37]. The purpose of gliosis is unclear and has been associated with both protective and detrimental effects ([38], reviewed in [14]). In the acute stage, upregulation of intermediate filaments is thought to play a beneficial role by facilitating the cytoarchitectural remodelling necessary for glial cells to protect the integrity of the tissue by limiting the lesion site and by modulating the basic neuroprotective function of glial cells. Conversely, prolonged upregulation can interfere with neuronal survival and regeneration. Following extensive loss of the photoreceptors within the ONL, the hypertrophied side branches of Müller cells may grow into the outer plexiform layer in an attempt to protect the inner retina from further degeneration. However, this action may also limit synaptic remodelling by the remaining neurons [39,40].

While variations in the timing and magnitude of response have been reported by various studies looking at different initiating insults, to our knowledge there have been no within- study comparative assessments of gliosis across time in multiple models of retinal degeneration. As anticipated, in our study, we found that the levels of Gfap and vimentin typically increased with disease progression (and age) in the majority of examined models, including wild-type animals. This upregulation was particularly striking in the *Rho* ^-/-^ model of retinitis pigmentosa, where Gfap^+ve^ Müller glial fibres extended beyond the ONL and along the outer margins of the retina, similar to the glial scars formed in the brain [41]. More surprising, however, was the repeated observation that the levels of both Gfap and vimentin, although increasing initially, undergo a regional decrease in the outer retina in both *Prph2* ^*+/Δ307*^ and *Pde6b* ^*rd1/rd1*^ animals at the later stages of degeneration. This is striking, when one considers that these models represent relatively slow and fast rates of degeneration, respectively. Conversely, the same phenomenon was not seen in the *Rho* ^-/-^ a model that falls somewhere between these two in terms of degeneration rate.

A reduction in Gfap was reported in the *Pde6b* ^*rd1/rd1*^ mouse, as assessed by IHC, at a stage mid-way between the time points examined here (P30) [42], while Roesch et al., performed a microarray data analysis from single Müller cells isolated from *Rho* ^-/-^ and *Pde6b* ^*rd1/rd1*^ neural retinae at two different stages of degeneration [43]. They reported that at 8 weeks, during the peak of rod death, four of five *Rho* ^-/-^ Müller cells expressed *Gfap* but only one of five of cells had significant levels of *Gfap* at 22 weeks, which corresponds to the peak of cone death. Since our histological data correlate with the 8 weeks findings reported in earlier by Roesch and colleagues [44], it may be that a reduction in Gfap levels occurs much later in degeneration in this model. However, we did not see this in additional samples taken from 22 weeks old mice, in which straining for Gfap remained very strong (S22A–S22F Fig.). Similarly, when Roesch et al., looked at the *Pde6b* ^*rd1/rd1*^ model, they found a robust expression of *Gfap* in four of five of the *Pde6b* ^*rd1/rd1*^ Müller cells at P12. We found little Gfap at the slightly earlier time point of P10, but robust levels by P15 (S23A–S23F Fig.). However, at 5 weeks, where Strettoi et al. [42] and our own study found a reduction in Gfap protein, Roesch and colleagues reported a significant expression of *Gfap* in five of five cells [44]. The apparent discrepancies almost certainly lie in the different techniques used. In the Roesch study, they used a powerful, single cell profiling approach, which reflects RNA levels at a given point in time while most studies including our own examined Gfap protein, which may undergo changes on a different timescale. In addition, significant heterogeneity among even wild-type Müller cells has been found in their previous study. Since cell death in the RP retina typically spreads from the centre to the periphery of the mouse retina, cells originating from the centre might differ significantly from those at the periphery. The data in the study by Roesch et al. necessarily come from only a very small number of cells (5 in each condition) and thus may not reflect the overall changes occurring in the diseased retina as a tissue.

Another reported feature of reactive gliosis associated with disease and injury is an increased deposition of proteoglycans, CSPGs being the predominant proteoglycan of the retina. CSPGs belong to a large family of glycoproteins that are found both on the cell surface and in the extracellular space. They consist of a core protein covalently attached to long carbohydrate chains known as glycosaminoglycans (GAGs). These GAG chains can vary markedly in length, number and sulphation pattern, which influence the unique functions of different proteoglycans within and between classes [45,46]. By interacting with other components of the ECM, proteoglycans play an important role in many biological processes, including cell development, migration and regulation of synaptic plasticity [12]. In the spinal cord and brain, CSPGs are upregulated by reactive astrocytes in response to injury and disease and participate in the inhibition of axon regeneration, mainly through their GAG side chains ([47,48], reviewed in [13]). However, our understanding of CSPG biosynthesis, and the role of these molecules in retinal degeneration, is limited. Here, we showed that CSPGs are found in several regions of the healthy neural retina including the GCL, IPL, OPL, and the IS/OS region. Taylor and colleagues reported alterations in the biosynthesis and turnover of proteoglycans with age [49], which is supported by our observations of an increase in CS-56 staining in aged (1 year), compared to young, wild-type mice. Importantly, others have linked age-related changes in the glycomatrix to the development of AMD pathogenesis [50,51]. Here, we report that in the degenerating retina CSPG levels usually, but not always, followed the trends in intermediate filament expression, becoming highly expressed in the *Rho* ^-/-^ model, but decreasing in the *Pde6b* ^*rd1/rd1*^ model.

Having identified marked but contrasting changes in the expression of CSPGs as assessed with the broad-spectrum CSPG marker, CS-56, further work is required to examine changes in specific CSPGs within the diseased retina. While a number of different proteoglycans have been identified, only a few have been studied in any detail in the eye. One that has received attention is neurocan (Ncan). *Ncan* expression is increased in retinae injured by transient ischemia, and in the *Pde6b* ^*rd1/rd1*^ mouse and Royal College of Surgeons (RCS) rat models of inherited retinal degeneration [4]. Interestingly, it has also been shown that neurocan inhibits the outgrowth of RGCs *in vitro*. This inhibition persists even after removal of chondroitin sulfate sugar chains from the core protein, suggesting that the neurocan core protein itself is inhibitory. Similarly, *Acan*, one of the major CSPGs expressed in nervous tissue [52,53], is expressed in the optic nerve and the inner retinal layers from embryonic day 16 (rat) and 18 (mouse) [54,55]. In development, aggrecan is produced by glial precursor cells and plays a significant role in neurite outgrowth [56,57] and astrocyte differentiation [58], amongst other roles. IHC analysis of the adult wild-type retina showed aggrecan in the GCL, INL, ONL and weak staining in the plexiform layers, which is similar to what has been shown in human retina [59].

The final feature we examined was the OLM, a network of heterotypic adherens junctions complexes that are linked to the cell skeleton [60]. These junctions help to maintain the orientation and polarity of the photoreceptors [61] and provide a semipermeable diffusion barrier for extracellular components [62]. Since these junctions are form between the inner segments of the photoreceptors and the apical processes of Müller cells, we reasoned that these would likely be affected by photoreceptor loss. In the wild-type mouse, the OLM was continuous throughout the entire retina at all time points examined. However, in each of the models of inherited retinal degeneration studied, there was evidence of OLM remodelling, although the nature and extent of this remodelling varied markedly between models. In addition, some photoreceptor cell bodies were mislocalized to the segment region. This observation correlates with previous studies in which chemically-induced swelling of Müller cells led to disruption of the OLM and photoreceptor cell nuclei mislocalization [27,63]. Surprisingly, however, OLM integrity was typically maintained even in severely degenerated retinae, demonstrating the importance of this barrier and the retina’s attempts to maintain it in the face of photoreceptor loss. Notably, with degeneration there is a shift in the proportion of junctions formed between Müller glia, compared to junctions between Müller glia and photoreceptors. Remarkably, in the *Rho* ^-/-^ model where the loss of the ONL occurs moderately quickly, the OLM seemed to stay linear and uninterrupted until at least 6 weeks. It was only at the latest time point examined that some disruption in the OLM was evident. In support of this observation, Zo-1 staining was readily detectable even at very late stage degeneration (22 weeks) with few interruptions (S22G–S22L Fig.). These observations contrast those of Campbell et al. [64,65], who reported reduced and disturbed Zo-1 staining as early as 3 weeks in *Rho* ^-/-^ mice and no staining at the OLM after 5 weeks. The reasons for this discrepancy are unclear. The *Crb1* ^*rd8/rd8*^ mouse is the major exception to this pattern. Loss of Crb1 leads to displaced photoreceptors and focal degeneration of all neural layers. This is attributed to loss of adhesion between photoreceptors and Müller cells [22,32], although more recent reports have indicated this phenomenon is background-specific [66]. As described by others, we found OLM integrity to be increasingly compromised with degeneration in this model [8,22]. In summary, disruption to the OLM might be an expected outcome of photoreceptor loss and would be expected to have significant consequences for the overall homeostasis of the retina [67]. Remarkably, however, our data show that while there is undoubtedly significant remodelling, the OLM very often remains intact even at advanced stages of degeneration.

It is increasingly evident that understanding the diseased retinal environment is of paramount importance when designing effective therapeutic strategies for the treatment of retinal degeneration. In the past few years, great progress has been made in the development of novel ocular therapies such as electronic implants [68] and in the field of both cell [6,69] and gene therapy [5,70,71]. Where therapeutic agents such as donor cells or viral vectors are injected in the subretinal space, gliosis may act as a physical and chemical barrier to their integration or penetration into the host retina. On the contrary, a compromised OLM may promote gene delivery or cell transplantation efficiency by increasing accessibility of the virus or the donor cell into the target tissue. Previously we have shown that by combining a reversible disruption of the OLM [8,27,28] with photoreceptor transplantation it is possible to improve levels of donor cell migration and integration into the recipient retina. Recently, Barber et al. showed that both OLM integrity and gliosis appear to play a role in cell transplantation efficiency; donor cell integration was poorest in murine models in which the integrity of the OLM is largely maintained and gliosis increases with disease progression, such as in *Rho* ^-/-^ mice [8]. Conversely, integration increases in the *Prph2* ^*+/Δ307*^ model, which, as shown here, undergoes significant remodelling of the OLM and also presents a reduction in Gfap levels at later stages of degeneration. Using transgenic animals lacking both Gfap and vimentin in Müller cells, Kinouchi and colleagues reported that these animals create a more permissive environment for subretinally grafted cells, compared to wild-type animals [26]. Cells transplanted into the double knockout retina showed better cellular integration as well as higher neurite outgrowth, compared with those transplanted into wild-type retina. However, since these double knock-out mice have also been reported to have disrupted limiting membranes and a prevalence for detachment and shearing [37], it is not possible to distinguish between the influence of the primary and secondary changes on the enhanced donor cell integration within the recipient retina. Confusingly, there is also evidence to suggest that gliosis can have a positive impact on cell transplantation. Nishida et al. reported in rat that upregulation of *Gfap* following retinal damage may promote the survival of transplanted neuronal stem cells; in regions with increased levels of Gfap, survival of transplanted stem cells was better in comparison with normal, undamaged retinas [72]. Together, these findings have led to a hypothesis that changes in the properties of the Müller glial cytoskeleton after injury may play a beneficial role in the migration and integration of transplanted cells, at least in the acute stage. In contrast, chronic upregulation of intermediate filament proteins may contribute to glial hypertrophy and formation of a glial scar, which may impede the integration of transplanted donor cells.

Gliosis has also been reported to have a detrimental effect on the application of viral vectors. Lentiviral vector is known to have better transduction efficiency in newborn photoreceptors and immature retinae than in adult mice after either intravitreal or subretinal injection [73–75] and Grüter and colleagues hypothesised that this might be because in adult mice the CSPG-rich interphotoreceptor matrix acts as a physical barrier [7]. By applying CSPG-digesting enzymes to the subretinal space in conjunction with lentiviral vector delivery, they obtained an improvement of viral diffusion and transduction of photoreceptors. It is interesting to note, however, that the transduction efficiency of lentivirus is still markedly lower than that achieved by adeno-associated virus (AAV) vectors without any treatment. One of the major differences between these two vectors is their viral particle size, ~20 nm for AAV vectors compare to ~80–100 nm for the lentiviral vector. The smaller particle size may facilitate the diffusion of the AAV vector through barriers such as the OLM and so enhance its access to the photoreceptor layer.

A more precise understanding of the profile of gliosis in different types of degeneration will enable the development of tailored strategies that prevent either the formation of the glial scar or break aspects of the scar down. For example, the bacterial enzyme chondroitinase ABC (ChABC), which breaks down CSPGs, has been applied to photoreceptor transplantation protocols with encouraging results. Numerous studies with both stem cell and photoreceptor precursor transplants have demonstrated that treatment with ChABC prior the transplantation increased the number of integrated cells [8,76,77]. Finally, combining cell transplantation with the manipulation of two or more barriers will be another interesting approach to investigate. Here, the spatiotemporal atlas of gliosis and OLM integrity changes in different degenerating retinae can be considered an important tool to use when designing future strategies to breakdown these barriers.

Our study shows that gliosis and the associated changes in CSPG deposition and OLM remodelling do not change in an equivalent manner between different degenerations and, importantly, are unrelated to disease severity. Here we examine only inherited causes of retinal degeneration and further studies are required to directly compare these with age-related pathology and injury-induced changes. It is also important to remember that the changes in Gfap, Vimentin, CSPGs and OLM integrity represent just a few changes in what is an incredibly complex event. Nonetheless, our findings underlie the need to study these processes and how they differ in various disease models. Such knowledge will be crucial to designing therapeutic strategies that will be required to circumvent these obstacles in the diseased retina.

## Supporting Information

S1 Table Antibodies and immunohistochemistry protocols used to assess markers of gliosis and OLM integrity. NGS: Normal goat serum (Abd serotec, Oxford UK). BSA: Bovine Serum Albumin (Sigma Aldrich, Dorset UK).
(DOC)

S2 Table Antibodies and protocols used for Western Blot analysis to assess expression of markers of gliosis. H2B: Histone 2B, PBST: PBS-Tween-20, HRP: Horseradish peroxide; BSA: Bovine Serum Albumin.
(DOC)

S1 Fig Comparison of three different housekeeping markers for Western Blot analysis in wild-type and *Pde6b* ^rd1/rd1^ animals. Levels of NL-68, Brn3b, and H2B were compared on the same gel across different ages in wild-type and *Pde6b* ^*rd1/rd1*^ animals (n = 3). After close consideration H2B was chosen as the loading control since, despite progressive degeneration, levels of this protein remained broadly constant across all time points and models examined, compared to other common control markers.
(TIF)

S2 Fig Assessment of intermediate filament protein Gfap at early (6 weeks), middle (6 months) and late (12 months) time points in wild-type mice. Cryosections were immunostained for glial cell marker Gfap (green) and counterstained with nuclei marker Hoechst 33342 (blue). Scale bar, 50 μm.
(TIF)

S3 Fig Assessment of intermediate filament protein vimentin at early (6 weeks), middle (6 months) and late (12 months) time points in wild-type mice. Cryosections were immunostained for glial cell marker vimentin (green) and counterstained with nuclei marker Hoechst 33342 (blue). Scale bar, 50 μm.
(TIF)

S4 Fig Assessment of chondroitin sulphate proteoglycans (CSPGs) deposition at early (6 weeks), middle (6 months) and late (12 months) time points in wild-type mice. Cryosections were immunostained for CSPGs (CS-56, red) and counterstained with nuclei marker Hoechst 33342 (blue). Scale bar, 50 μm.
(TIF)

S5 Fig Assessment of the OLM integrity at early (6 weeks), middle (6 months) and late (12 months) time points in wild-type mice. Cryosections were immunostained for Zo-1 (red) and counterstained with nuclei marker Hoechst 33342 (blue). Scale bar, 25 μm.
(TIF)

S6 Fig Assessment of Gfap at early (3 weeks), middle (6 weeks) and late (12 weeks) time points in *Crb1* ^*rd8/rd8*^ model. Cryosections were immunostained for glial cell marker Gfap (green) and counterstained with nuclei marker Hoechst 33342 (blue). Scale bar, 50 μm.
(TIF)

S7 Fig Assessment of vimentin at early (3 weeks), middle (6 weeks) and late (12 weeks) time points in *Crb1* ^*rd8/rd8*^ model. Cryosections were immunostained for glial cell marker vimentin (green) and counterstained with nuclei marker Hoechst 33342 (blue). Scale bar, 50 μm.
(TIF)

S8 Fig Assessment of chondroitin sulphate proteoglycans (CSPGs) deposition at early (3 weeks), middle (6 weeks) and late (12 weeks) time points in *Crb1* ^*rd8/rd8*^ model. Cryosections were immunostained for CSPGs (CS-56, red) and counterstained with nuclei marker Hoechst 33342 (blue). Scale bar, 50 μm.
(TIF)

S9 Fig Assessment of the OLM integrity at early (3 weeks), middle (6 weeks) and late (12 weeks) time points in *Crb1* ^*rd8/rd8*^ model. Cryosections were immunostained for Zo-1 (red) and counterstained with nuclei marker Hoechst 33342 (blue). Scale bar, 25 μm.
(TIF)

S10 Fig Assessment of Gfap at early (2 months), middle (4 months) and late (6 months) time points in *Prph2* ^+/*Δ307*^ model. Cryosections were immunostained for glial cell marker Gfap (green) and counterstained with nuclei marker Hoechst 33342 (blue). Scale bar, 50 μm.
(TIF)

S11 Fig Assessment of vimentin at early (2 months), middle (4 months) and late (6 months) time points in *Prph2* ^+/*Δ307*^ model. Cryosections were immunostained for glial cell marker vimentin (green) and counterstained with nuclei marker Hoechst 33342 (blue). Scale bar, 50 μm.
(TIF)

S12 Fig Assessment of chondroitin sulphate proteoglycans (CSPGs) deposition at early (2 months), middle (4 months) and late (6 months) time points in *Prph2* ^+/*Δ307*^ model. Cryosections were immunostained for CSPGs (CS-56, red) and counterstained with nuclei marker Hoechst 33342 (blue). Scale bar, 50 μm.
(TIF)

S13 Fig Assessment of the OLM integrity at early (2 months), middle (4 months) and late (6 months) time points in *Prph2* ^+/*Δ307*^ model. Cryosections were immunostained for Zo-1 (red) and counterstained with nuclei marker Hoechst 33342 (blue). Scale bar, 25 μm.
(TIF)

S14 Fig Assessment of Gfap at early (4 weeks), middle (6 weeks) and late (10 weeks) time points in *Rho* ^-/-^ model. Cryosections were immunostained for glial cell marker Gfap (green) and counterstained with nuclei marker Hoechst 33342 (blue). Scale bar, 50 μm.
(TIF)

S15 Fig Assessment of vimentin at early (4 weeks), middle (6 weeks) and late (10 weeks) time points in *Rho* ^-/-^ model. Cryosections were stained with glial cell marker vimentin (green) and counterstained with nuclei marker Hoechst 33342 (blue). Scale bar, 50 μm.
(TIF)

S16 Fig Assessment of glial chondroitin sulphate proteoglycans (CSPGs) deposition at early (4 weeks), middle (6 weeks) and late (10 weeks) time points in *Rho* ^-/-^ model. Cryosections were immunostained for CSPGs (CS-56, red) and counterstained with nuclei marker Hoechst 33342 (blue). Scale bar, 50 μm.
(TIF)

S17 Fig Assessment of the OLM integrity at early (4 weeks), middle (6 weeks) and late (10 weeks) time points in *Rho* ^-/-^ model. Cryosections were immunostained for Zo-1 (red) and counterstained with nuclei marker Hoechst 33342 (blue). Scale bar, 25 μm.
(TIF)

S18 Fig Assessment of Gfap at early (P10, immature retina), middle (3 weeks) and late (6–7 weeks) time points in *Pde6b* ^rd1/rd1^ model. Cryosections were immunostained for glial cell marker Gfap (green) and counterstained with nuclei marker Hoechst 33342 (blue). Scale bar, 50 μm.
(TIF)

S19 Fig Assessment of vimentin at early (P10, immature retina), middle (3 weeks) and late (6–7 weeks) time points in *Pde6b* ^rd1/rd1^ model. Cryosections were immunostained for glial cell marker vimentin (green) and counterstained with nuclei marker Hoechst 33342 (blue). Scale bar, 50 μm.
(TIF)

S20 Fig Assessment of glial chondroitin sulphate proteoglycans (CSPGs) deposition at early (P10, immature retina), middle (3 weeks) and late (6–7 weeks) time points in *Pde6b* ^rd1/rd1^ model. Cryosections were immunostained for CSPGs (CS-56, red) and nuclei marker (blue). Scale bar, 50 μm.
(TIF)

S21 Fig Assessment of the OLM integrity at early (P10, immature retina), middle (3 weeks) and late (7 weeks) time points in *Pde6b* ^rd1/rd1^ model. Cryosections were immunostained for Zo-1 (red) and counterstained with nuclei marker Hoechst 33342 (blue). Scale bar, 25 μm.
(TIF)

S22 Fig Assessment of glial scarring and OLM integrity at 22 weeks old in *Rho* ^-/-^. **(A-F)** At this age, high levels of Gfap were present throughout the Müller glia processes. At the outer edge of the retina, Gfap^+ve^ processes were seen surrounding the remaining photoreceptor nuclei. **(G-L)** At this stage of degeneration, most of the photoreceptors are lost but the OLM remains largely intact. Cryosections were immunostained for glial cell marker Gfap (green) or Zo-1 (red) and co-stained with nuclei marker Hoechst 33342 (blue). Scale bar, 50 μm.
(TIF)

S23 Fig Assessment of gliosis and OLM integrity at P15 in *Pde6b* ^rd1/rd1^. **(A-F)** In comparison to P10 retina, there was a marked upregulation in Gfap protein with glial processes extending towards the ONL. **(G-L)** Similarly, more vimentin^+ve^ processes were observed extending towards the ONL. **(M-S)** At this stage, CSPGs were abundant in the subretinal space and the outer plexiform layer, but were reduced in the subretinal space in comparison to P10. **(T-Y)** In comparison to P10 animals, the OLM appeared more disrupted especially at the posterior regions. Cryosections were immunostained for glial cell marker Gfap (red) or vimentin (green) or CSPGs (red) and co-stained with nuclei marker Hoechst 33342 (blue). Scale bar, 50 μm.
(TIF)
