# Supplementary material for: Müller Glia Activation in Response to Inherited Retinal Degeneration Is Highly Varied and Disease-Specific
Source: PLoS One. 2015 Mar 20;10(3):e0120415. doi: 10.1371/journal.pone.0120415 (PMC4368159; doi:10.1371/journal.pone.0120415)
Supplement: S1 Table — NGS: Normal goat serum (Abd serotec, Oxford UK). BSA: Bovine Serum Albumin (Sigma Aldrich, Dorset UK). (DOC) [file pone.0120415.s001.doc]

| Antibody | Fixation | Blocking solution | Primary Ab concentration | Secondary Ab |
| --- | --- | --- | --- | --- |
| Polyclonal rabbit anti-Gfap  (DAKO; Z0334) | No fixation,  1% PFA post-fix (5 mins) | 2% NGS,  1% BSA  0.05% Triton-X  PBS | 1:500 | Gt-anti-rabbit  Alexa 488,  1:500; Molecular Probes |
| Monoclonal mouse anti-Vimentin (SIGMA V5255) | No fixation.  1% PFA post-fix (5 mins) | 5% NGS  2.5% BSA  0.05% Triton-X  PBS | 1:200 | Gt-anti-Mouse  Alexa 488,  1:500; Molecular Probes |
| Monoclonal mouse anti-CS56 (SIGMA C8035) | No fixation.  1% PFA post-fix (5 mins) | 1% NGS  5% Milk  0.05% Triton-X  PBS | 1:200 | Gt-anti-Mouse  Alexa 546,  1:500; Molecular Probes |
| Polyclonal rabbit anti-Zo-1 (Zymed) | No fixation.  1% PFA post-fix (5 mins) | 5% NGS  3% BSA  0.05% Triton-X  PBS | 1:250 | Gt-anti-rabbit  Alexa 546,  1:500; Molecular Probes |
