# Supplementary material for: Müller Glia Activation in Response to Inherited Retinal Degeneration Is Highly Varied and Disease-Specific
Source: PLoS One. 2015 Mar 20;10(3):e0120415. doi: 10.1371/journal.pone.0120415 (PMC4368159; doi:10.1371/journal.pone.0120415)
Supplement: S2 Table — H2B: Histone 2B, PBST: PBS-Tween-20, HRP: Horseradish peroxide; BSA: Bovine Serum Albumin. (DOC) [file pone.0120415.s002.doc]

| Antibody | Laemmli’s Buffer | Blocking solution (BS) | Primary Ab concentration | Secondary Ab |
| --- | --- | --- | --- | --- |
| Monoclonal mouse anti-H2B (Biolabs, #2934) | Reducing | 5% Non-fat milk  1% BSA  0.05% PBST | 1:10000 in  5% BSA  0.05% PBS-Tween (PBST) | Gt-anti-mouse conjugated to HRP, Perbio  1: 10000 in BS |
| Polyclonal rabbit anti-Gfap  (DAKO; Z0334) | Reducing | 5% Non-fat milk  1% BSA  0.05% PBST | 1:2500 in BS | Gt-anti-rabbit conjugated to HRP, Pierce  1: 5000 in BS |
| Monoclonal mouse anti- Vimentin (Sigma, V5255) | Reducing | 5% Non-fat milk  1% BSA  0.05% PBST | 1:3000 in BS | Gt-anti-mouse conjugated to HRP, Perbio  1: 10000 in BS |
| Monoclonal mouse anti-CS56 (SIGMA C8035) | Non-reducing | 10% Non-fat milk  0.05% PBST | 1:1500 in  5% Non-fat milk  0.05% PBST | Gt-anti-mouse conjugated to HRP, Perbio  1: 10000 in BS |
