# Supplementary figures and images for: Müller Glia Activation in Response to Inherited Retinal Degeneration Is Highly Varied and Disease-Specific
Source: PLoS One. 2015 Mar 20;10(3):e0120415. doi: 10.1371/journal.pone.0120415 (PMC4368159; doi:10.1371/journal.pone.0120415)

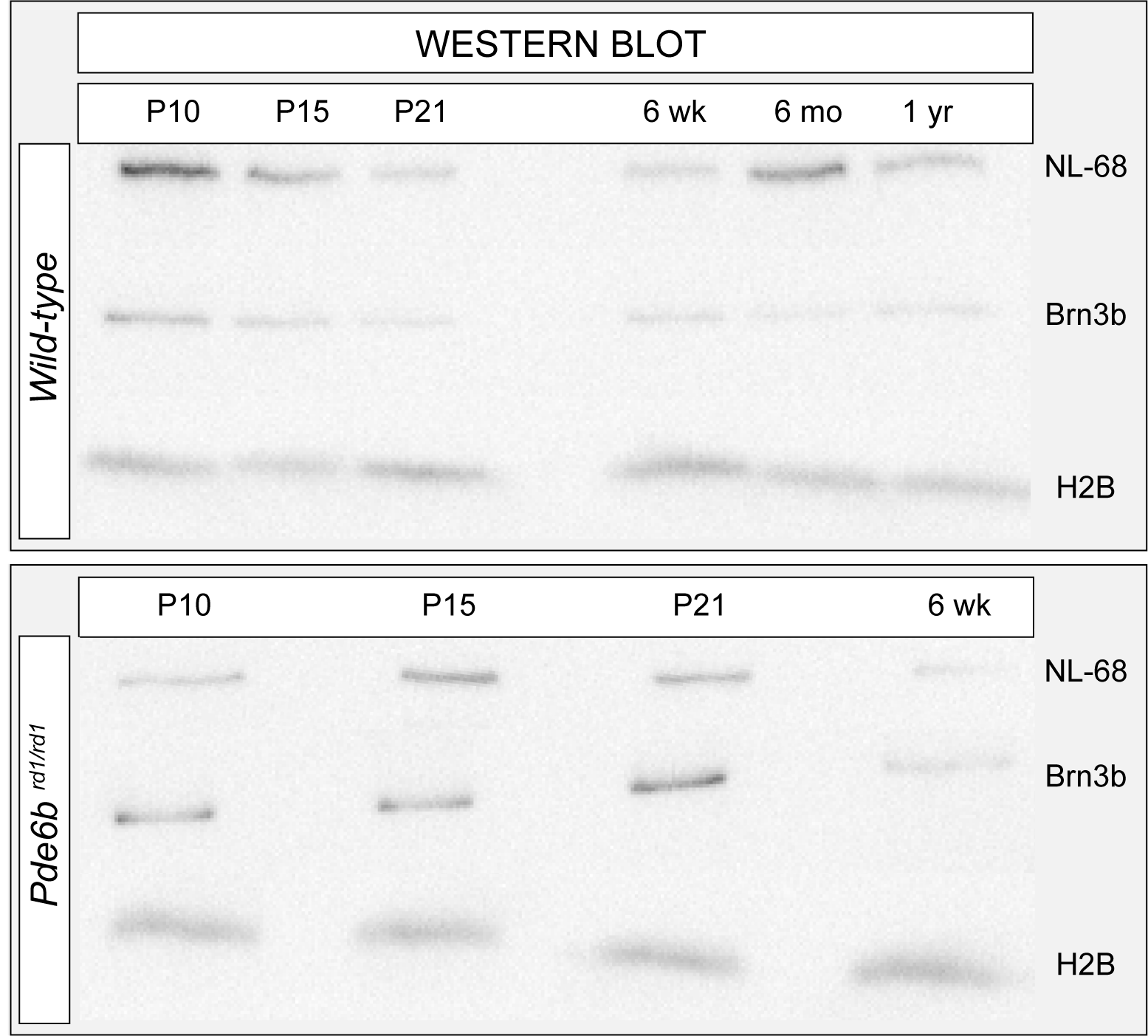

Supplement: S1 Fig — Levels of NL-68, Brn3b, and H2B were compared on the same gel across different ages in wild-type and Pde6b rd1/rd1 animals (n = 3). After close consideration H2B was chosen as the loading control since, despite progressive degeneration, levels of this protein remained broadly constant across all time points and models examined, compared to other common control markers. (TIF) [file pone.0120415.s003.tif]

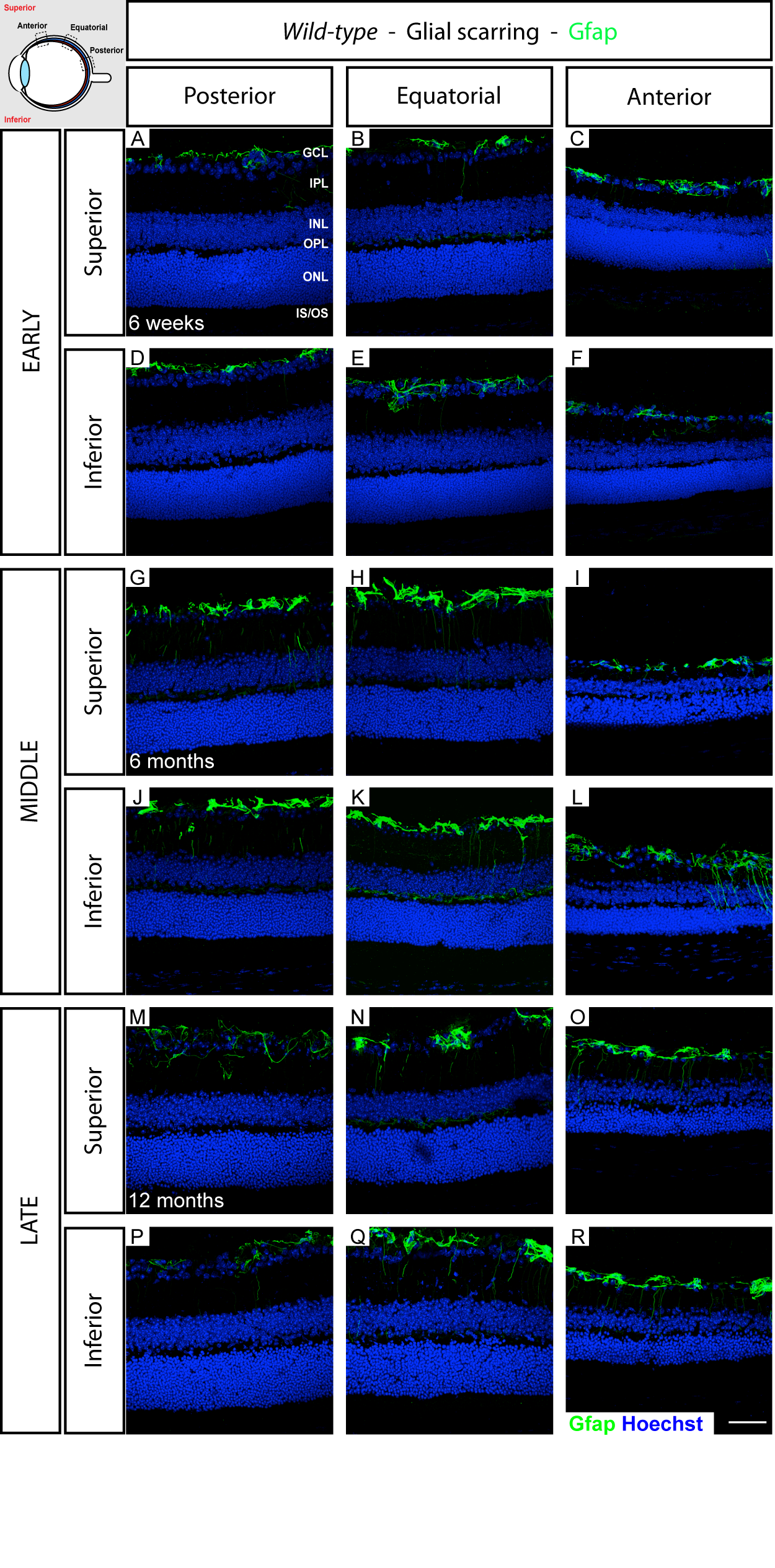

Supplement: S2 Fig — Cryosections were immunostained for glial cell marker Gfap (green) and counterstained with nuclei marker Hoechst 33342 (blue). Scale bar, 50 μm. (TIF) [file pone.0120415.s004.tif]

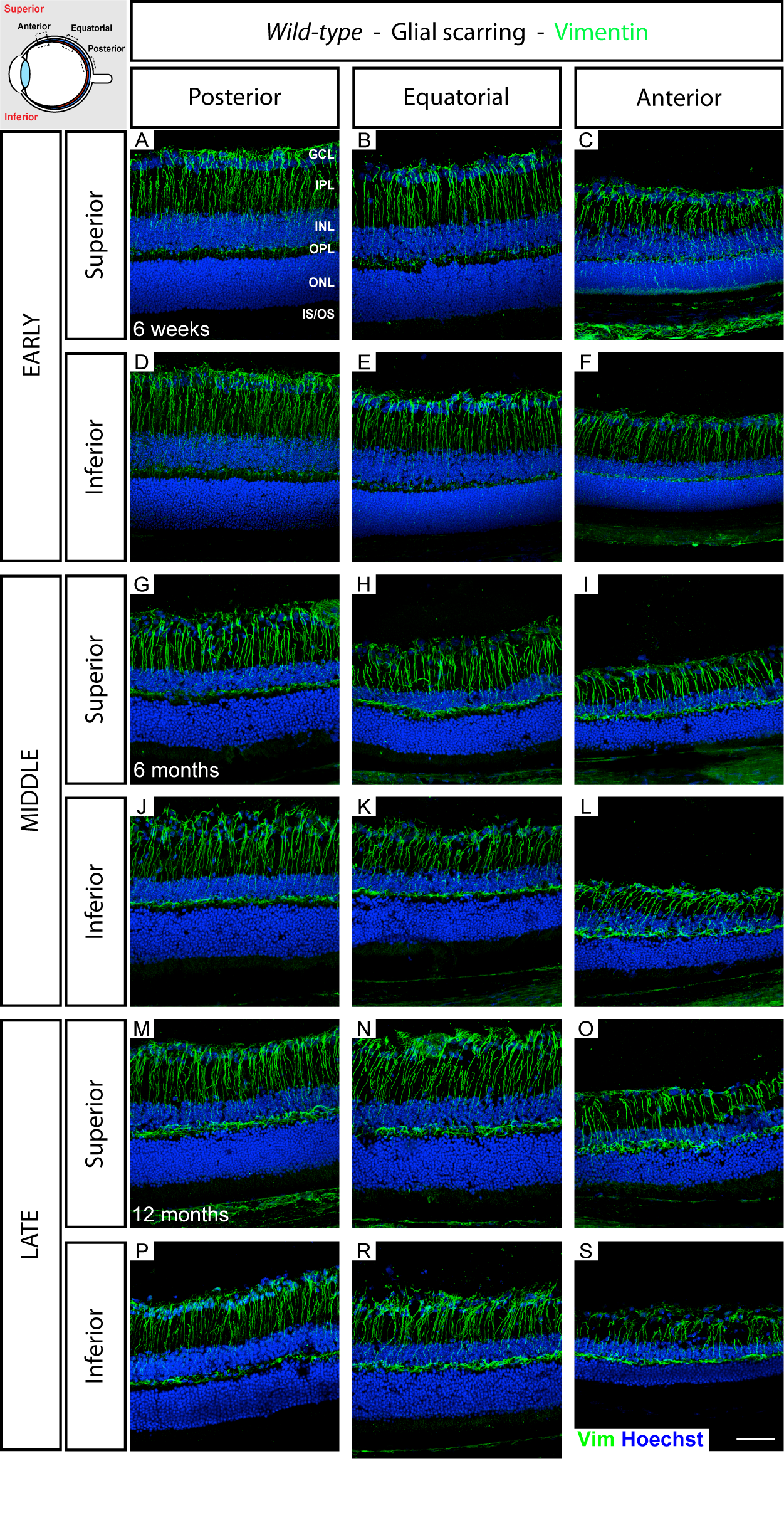

Supplement: S3 Fig — Cryosections were immunostained for glial cell marker vimentin (green) and counterstained with nuclei marker Hoechst 33342 (blue). Scale bar, 50 μm. (TIF) [file pone.0120415.s005.tif]

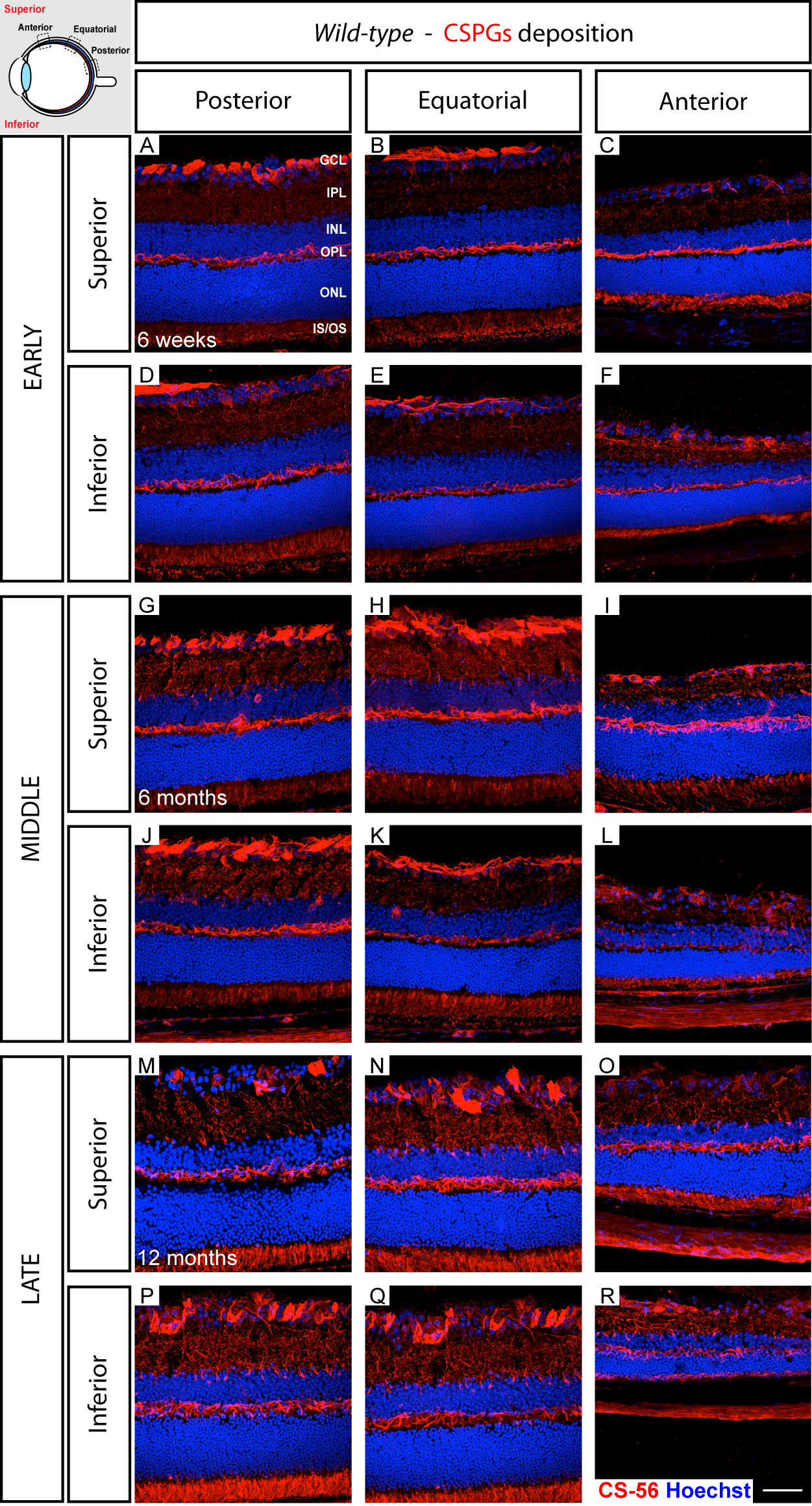

Supplement: S4 Fig — Cryosections were immunostained for CSPGs (CS-56, red) and counterstained with nuclei marker Hoechst 33342 (blue). Scale bar, 50 μm. (TIF) [file pone.0120415.s006.tif]

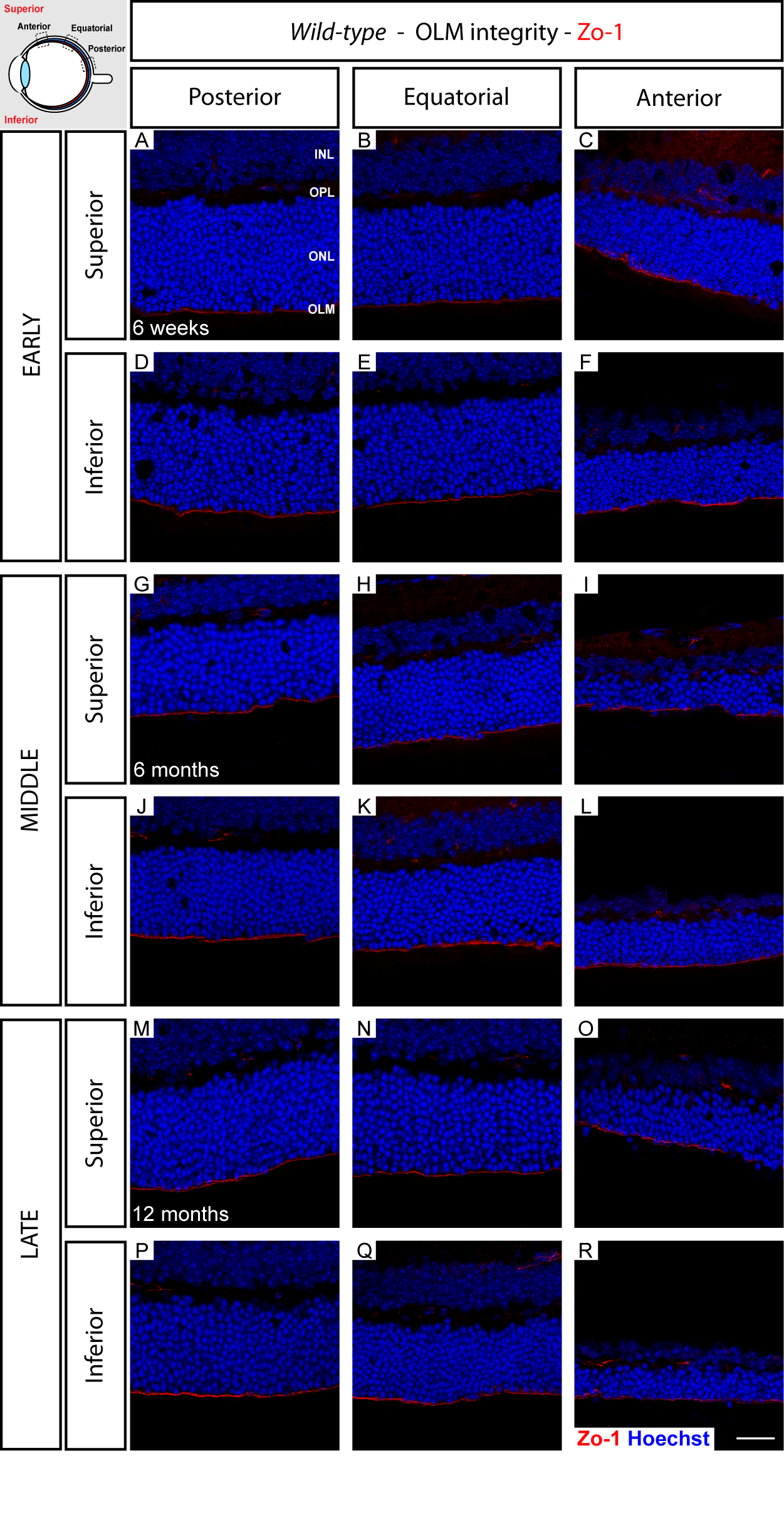

Supplement: S5 Fig — Cryosections were immunostained for Zo-1 (red) and counterstained with nuclei marker Hoechst 33342 (blue). Scale bar, 25 μm. (TIF) [file pone.0120415.s007.tif]

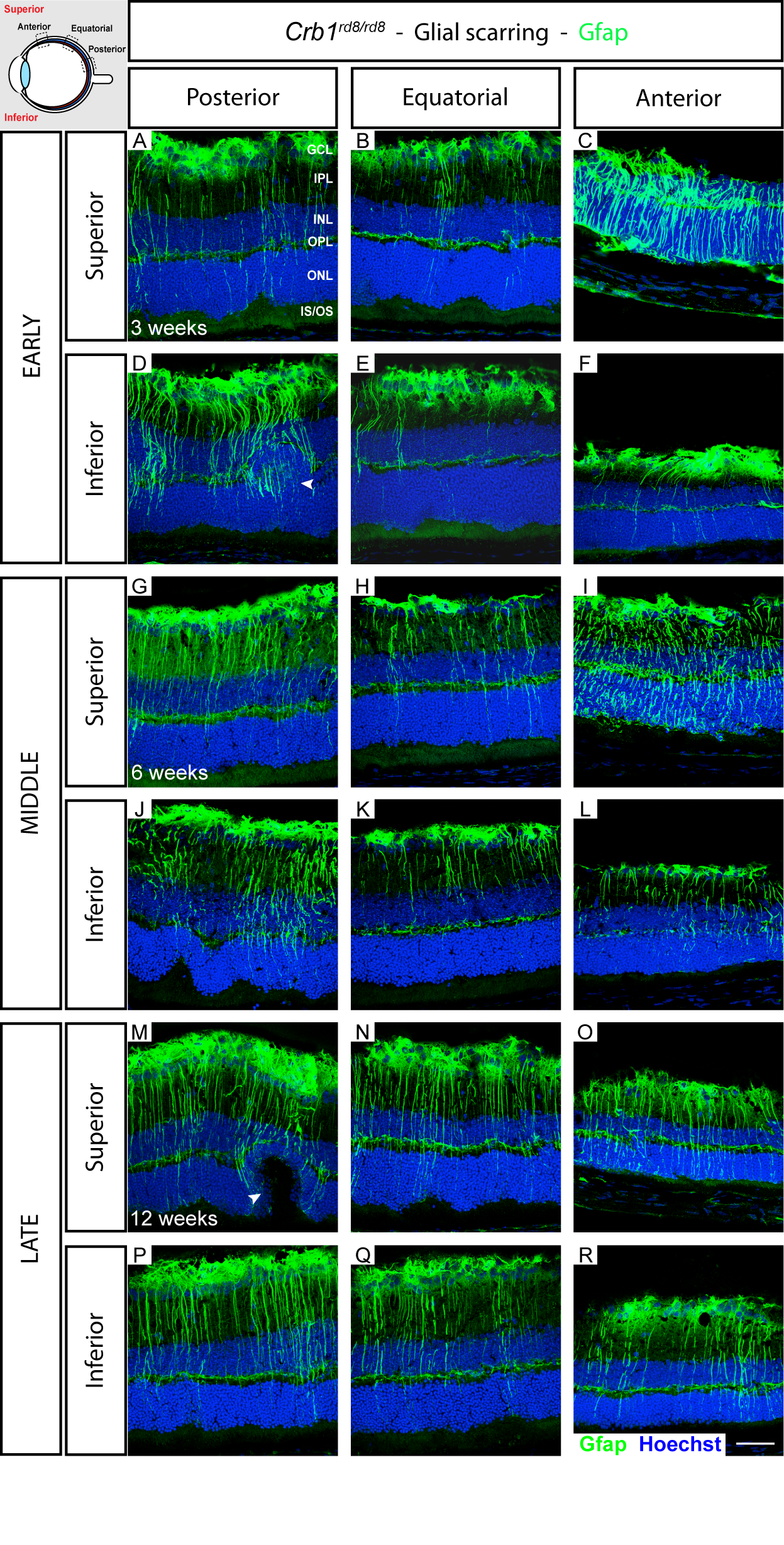

Supplement: S6 Fig — Cryosections were immunostained for glial cell marker Gfap (green) and counterstained with nuclei marker Hoechst 33342 (blue). Scale bar, 50 μm. (TIF) [file pone.0120415.s008.tif]

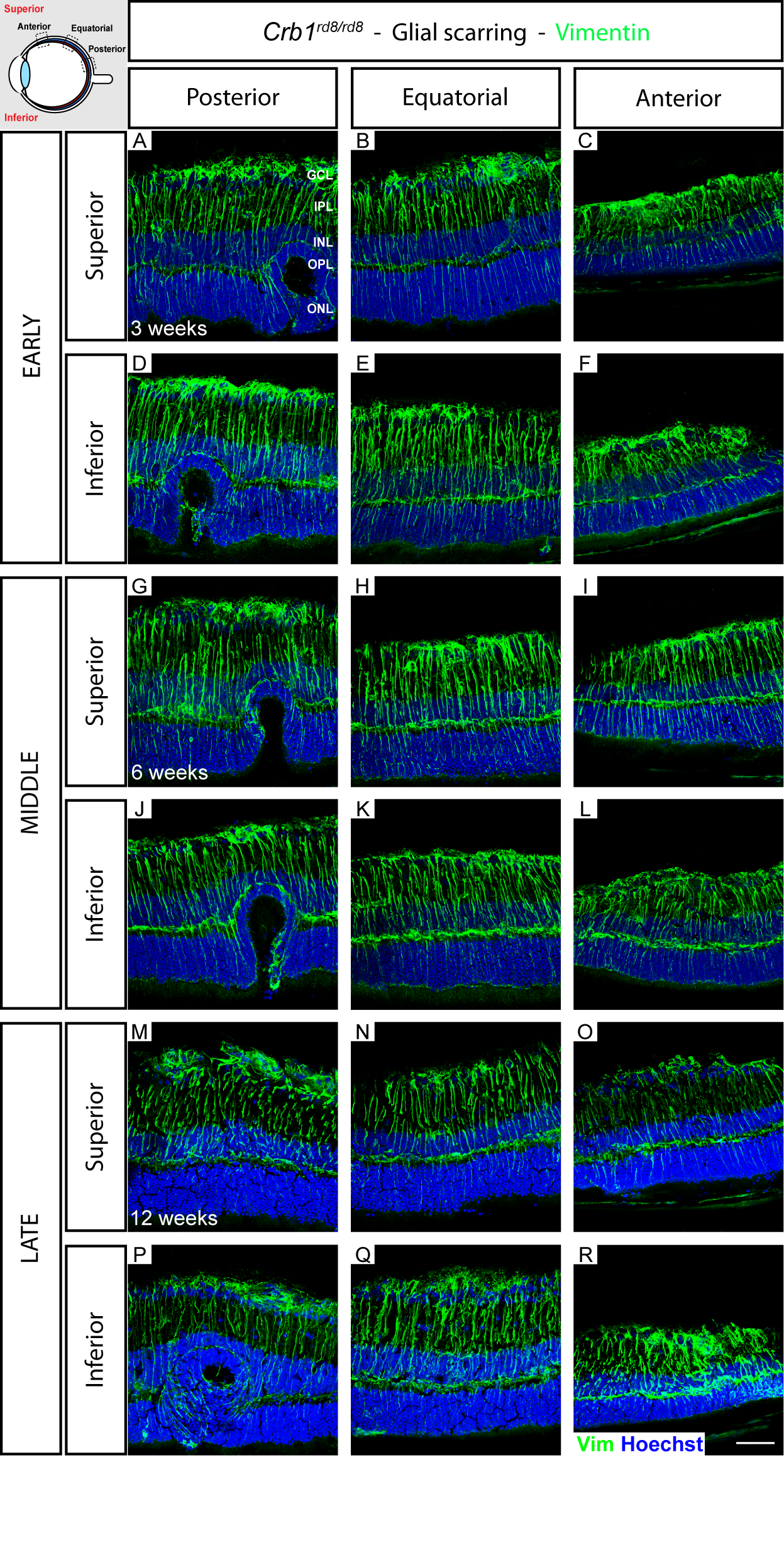

Supplement: S7 Fig — Cryosections were immunostained for glial cell marker vimentin (green) and counterstained with nuclei marker Hoechst 33342 (blue). Scale bar, 50 μm. (TIF) [file pone.0120415.s009.tif]

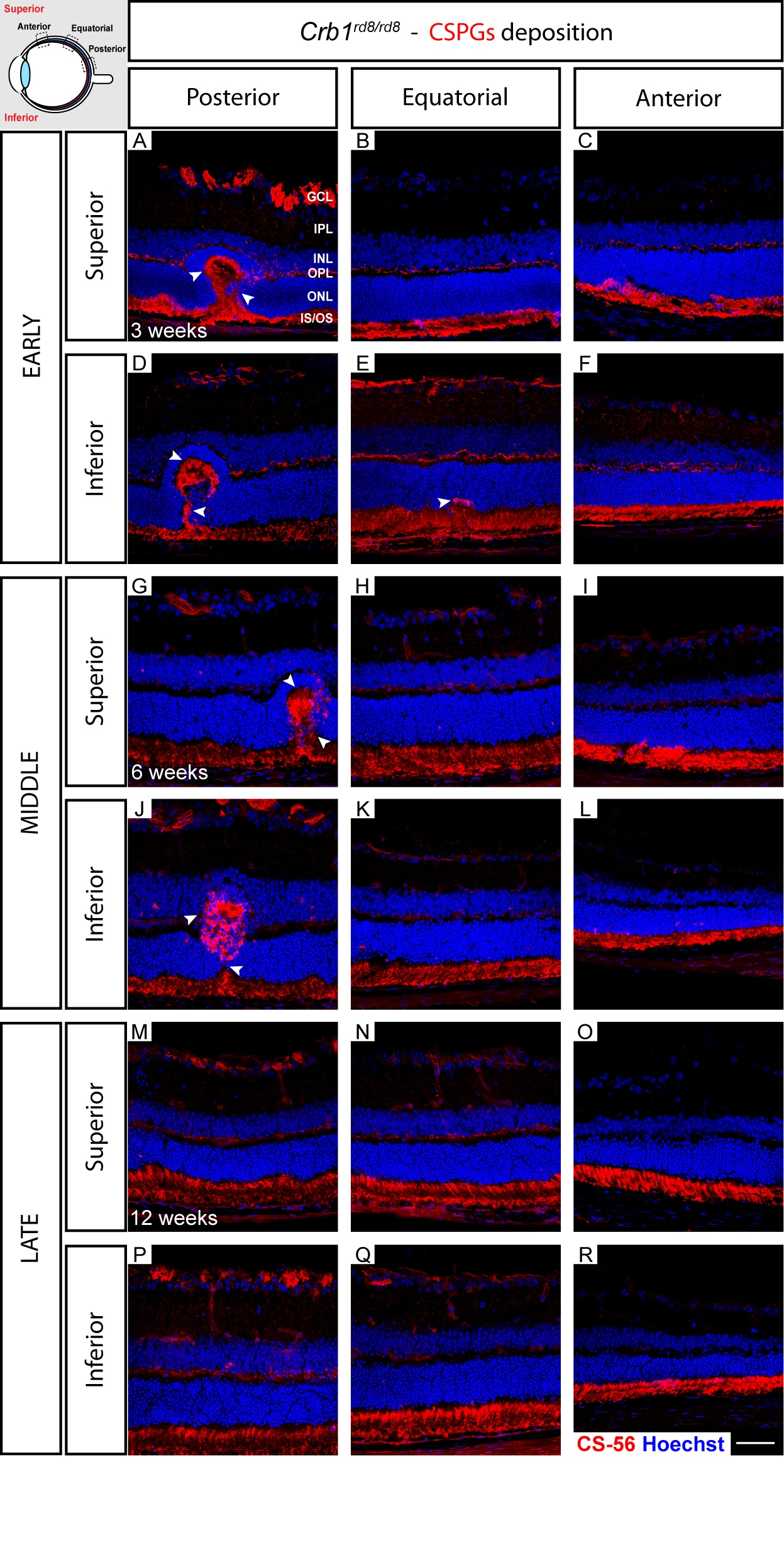

Supplement: S8 Fig — Cryosections were immunostained for CSPGs (CS-56, red) and counterstained with nuclei marker Hoechst 33342 (blue). Scale bar, 50 μm. (TIF) [file pone.0120415.s010.tif]

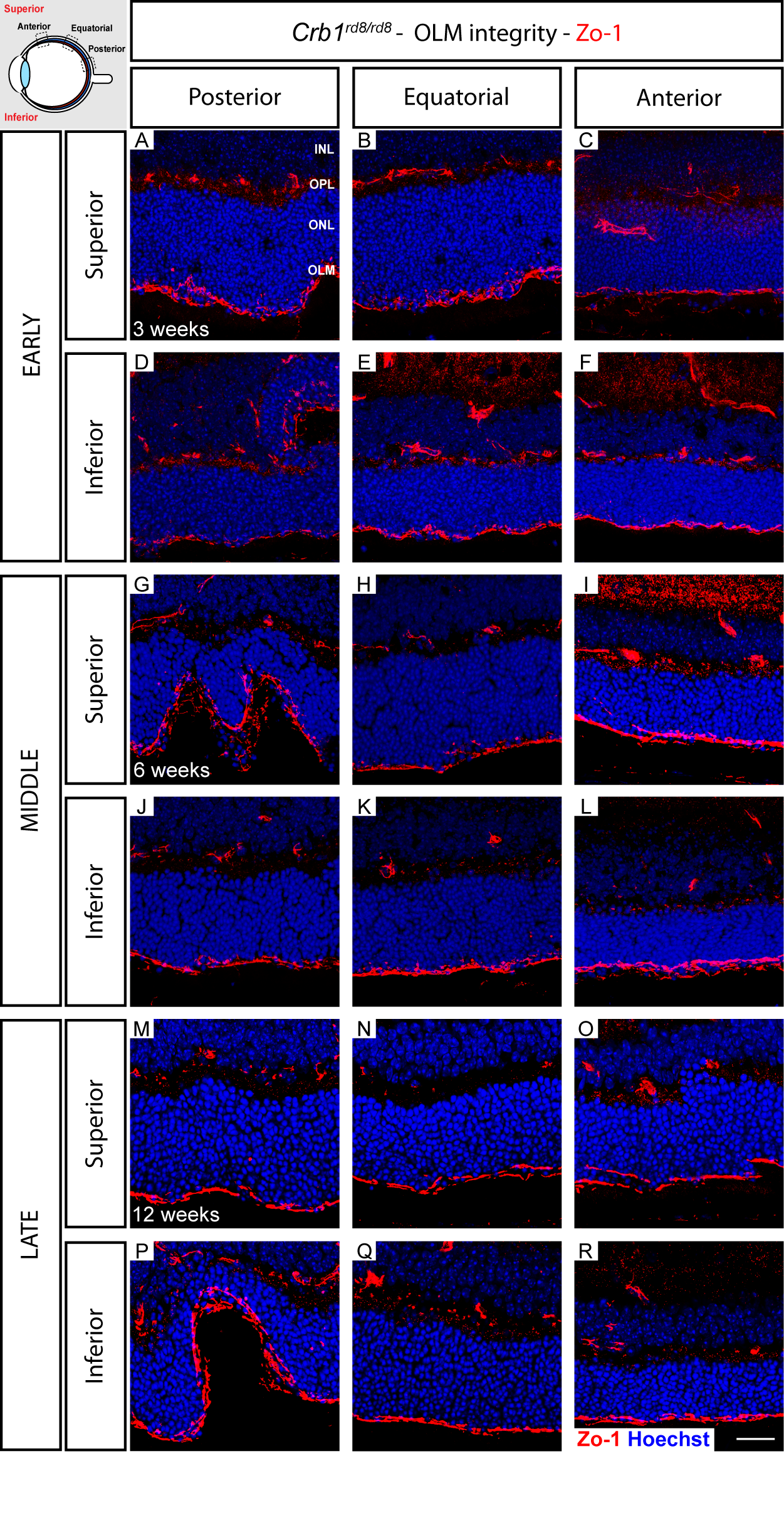

Supplement: S9 Fig — Cryosections were immunostained for Zo-1 (red) and counterstained with nuclei marker Hoechst 33342 (blue). Scale bar, 25 μm. (TIF) [file pone.0120415.s011.tif]

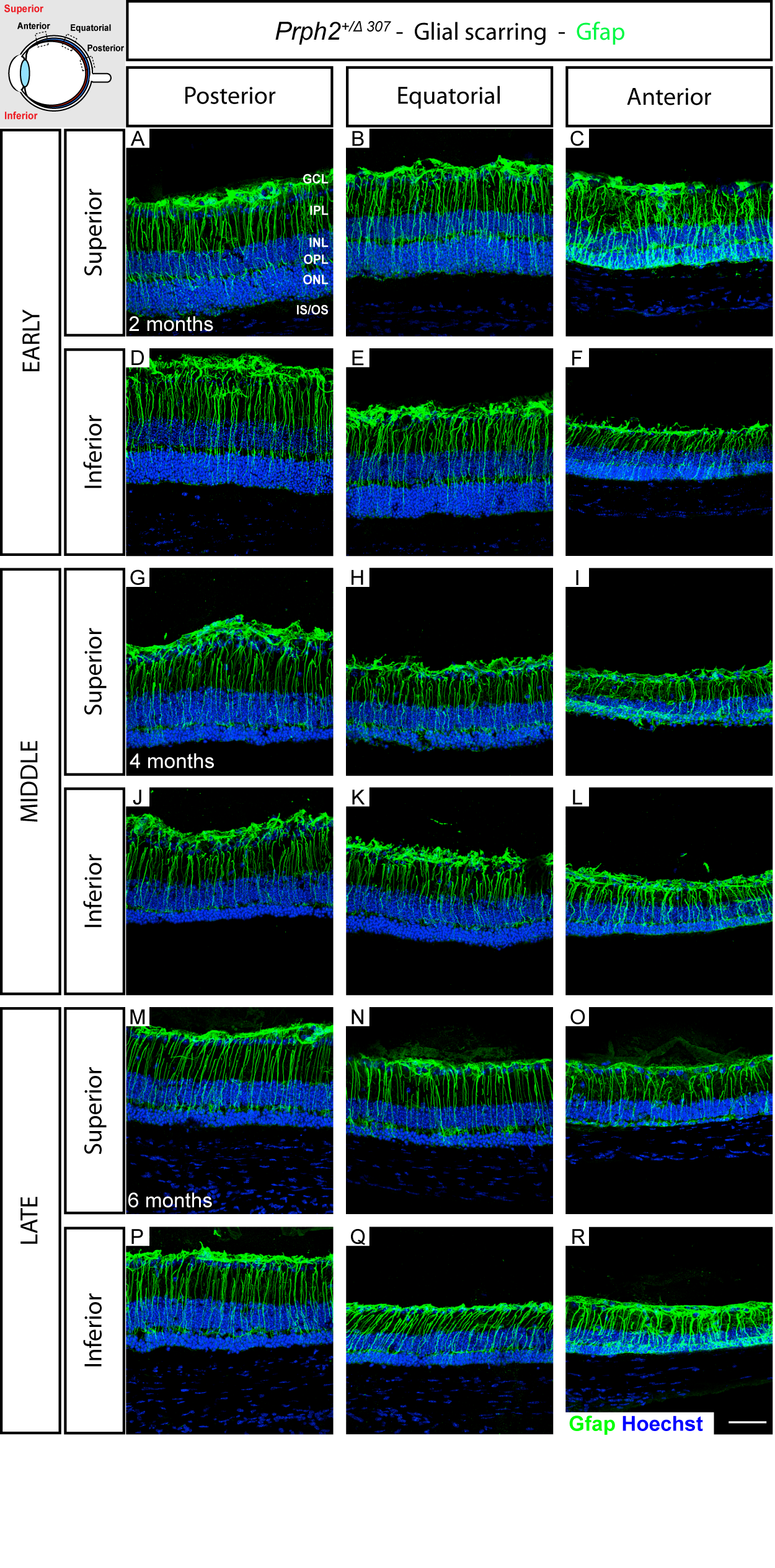

Supplement: S10 Fig — Cryosections were immunostained for glial cell marker Gfap (green) and counterstained with nuclei marker Hoechst 33342 (blue). Scale bar, 50 μm. (TIF) [file pone.0120415.s012.tif]

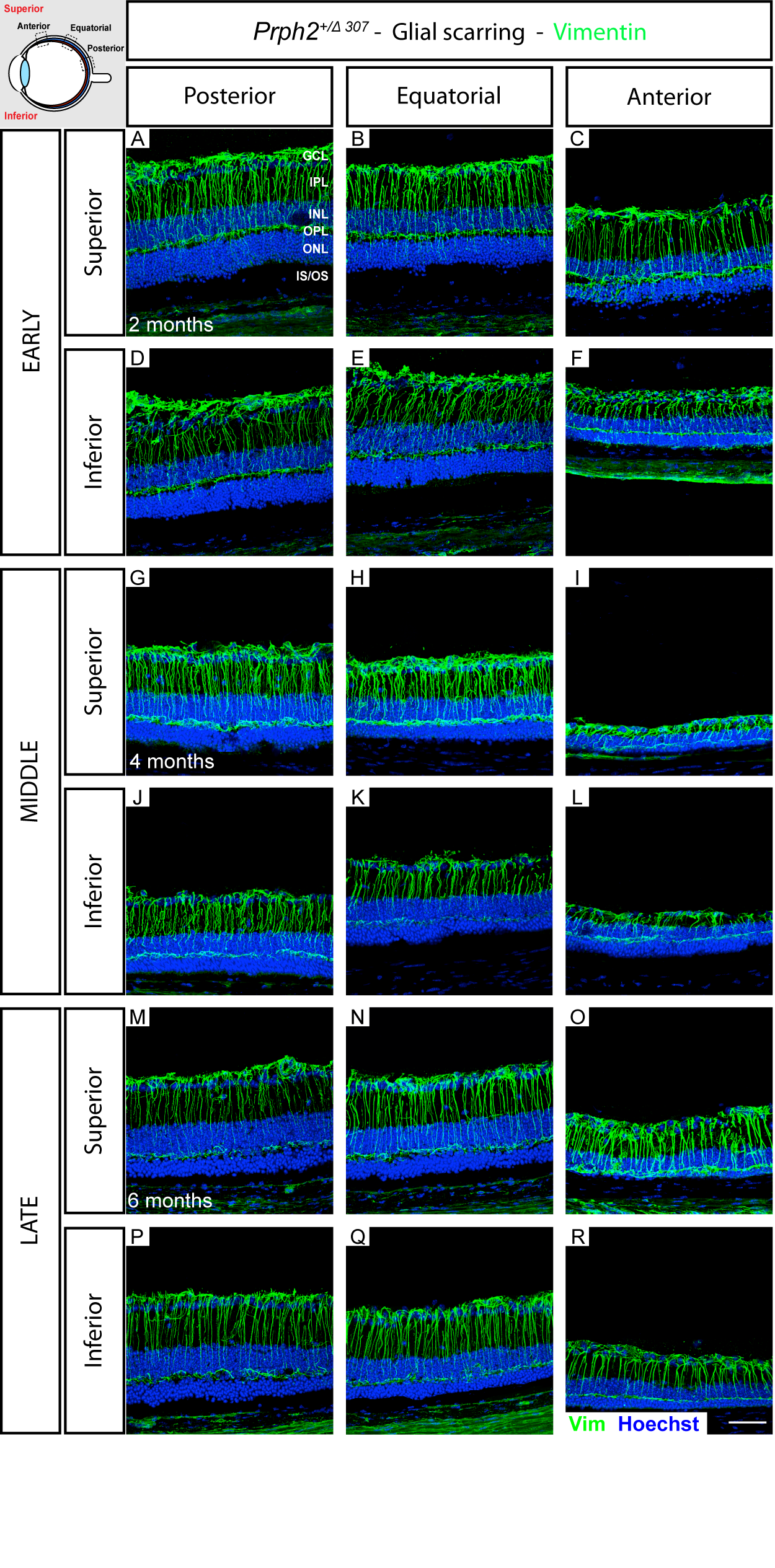

Supplement: S11 Fig — Cryosections were immunostained for glial cell marker vimentin (green) and counterstained with nuclei marker Hoechst 33342 (blue). Scale bar, 50 μm. (TIF) [file pone.0120415.s013.tif]

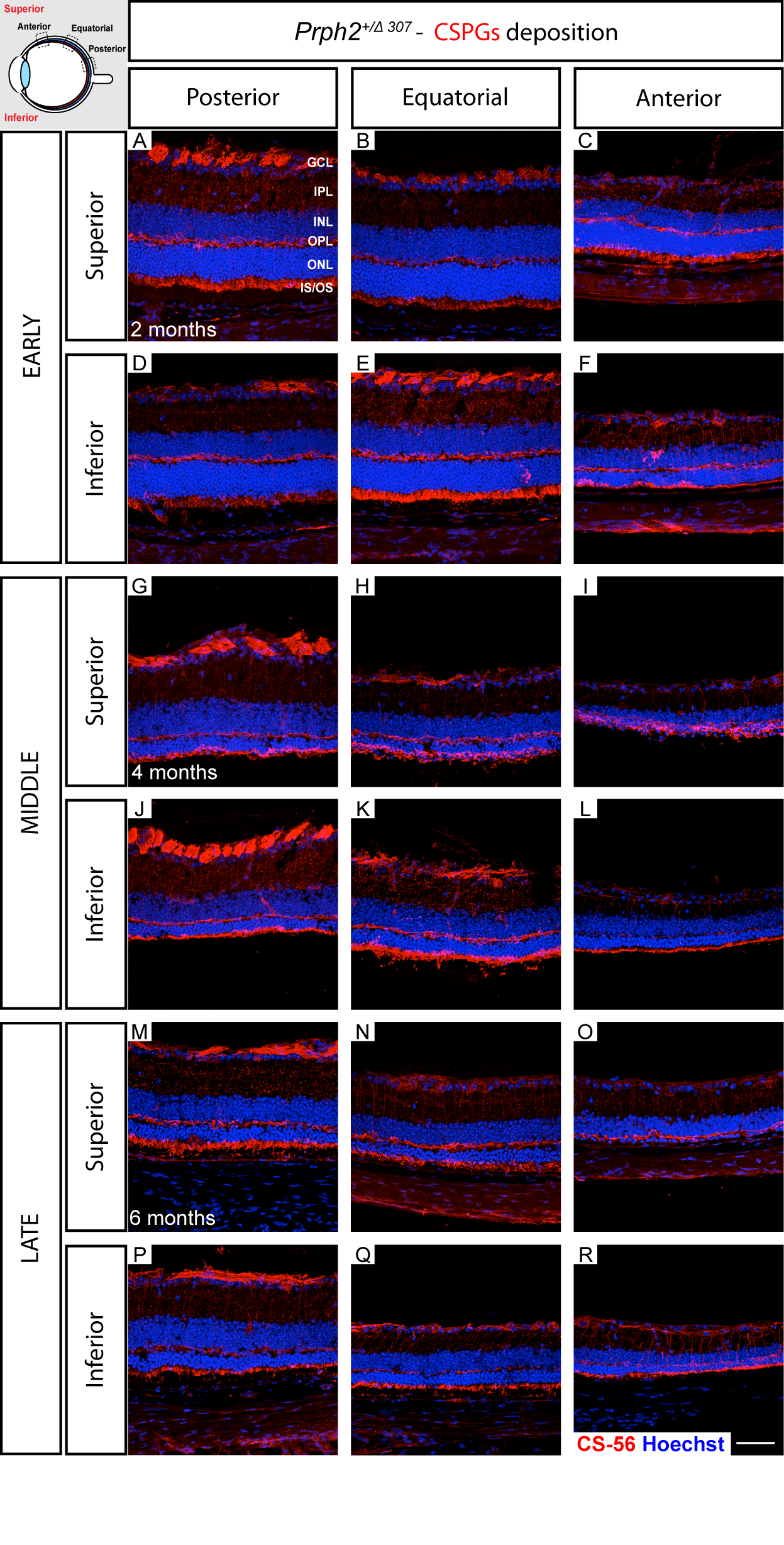

Supplement: S12 Fig — Cryosections were immunostained for CSPGs (CS-56, red) and counterstained with nuclei marker Hoechst 33342 (blue). Scale bar, 50 μm. (TIF) [file pone.0120415.s014.tif]

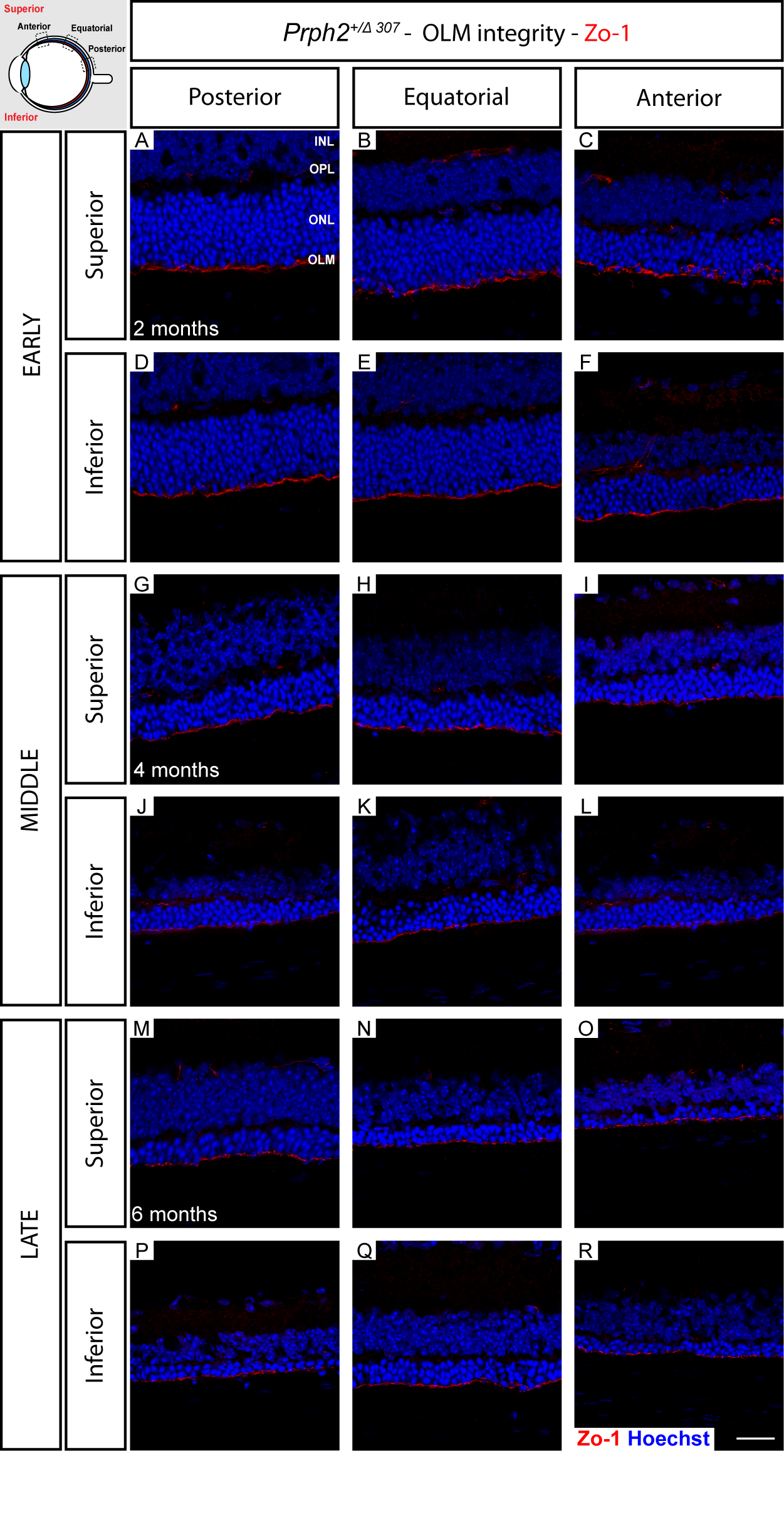

Supplement: S13 Fig — Cryosections were immunostained for Zo-1 (red) and counterstained with nuclei marker Hoechst 33342 (blue). Scale bar, 25 μm. (TIF) [file pone.0120415.s015.tif]

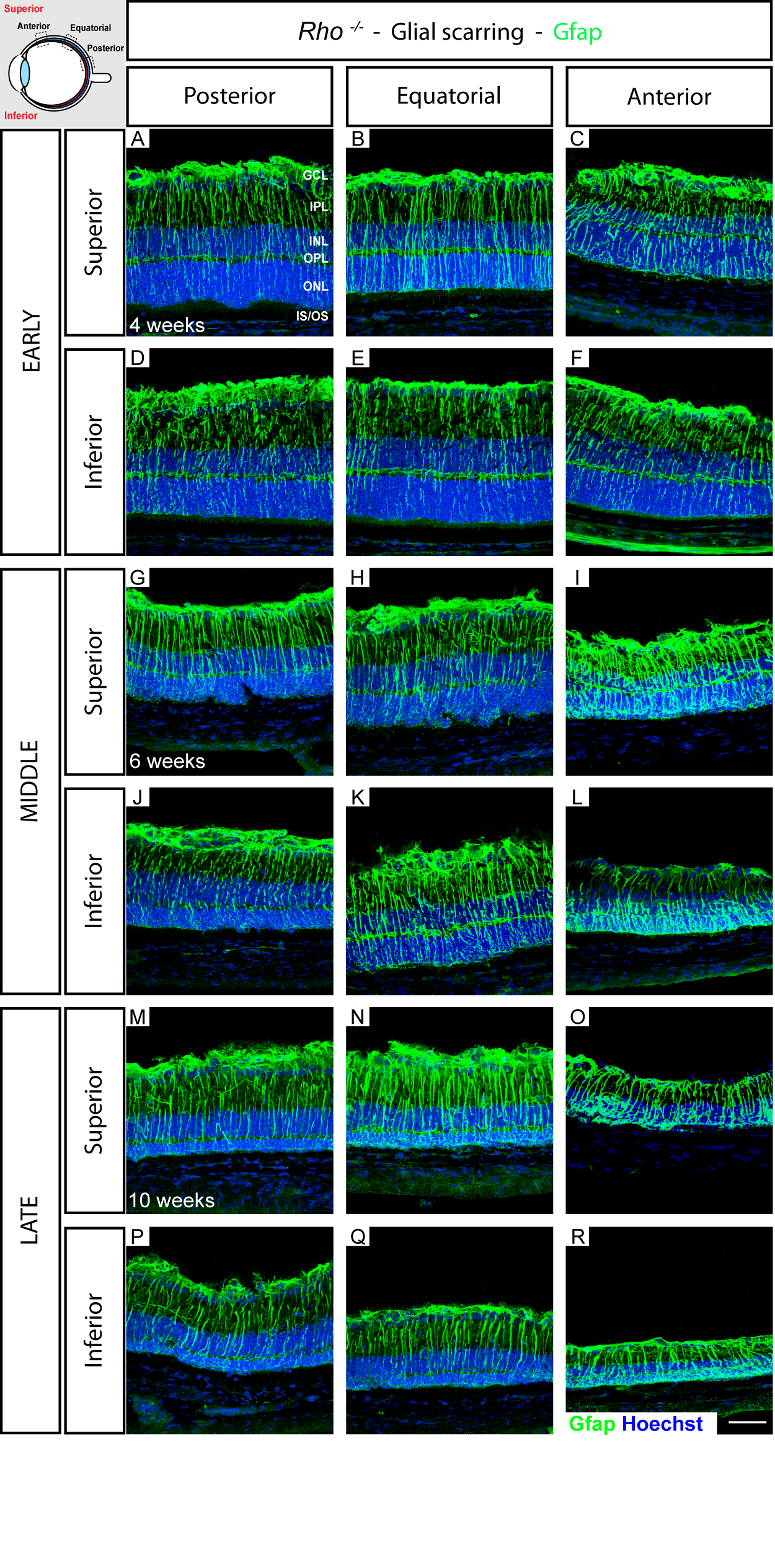

Supplement: S14 Fig — Cryosections were immunostained for glial cell marker Gfap (green) and counterstained with nuclei marker Hoechst 33342 (blue). Scale bar, 50 μm. (TIF) [file pone.0120415.s016.tif]

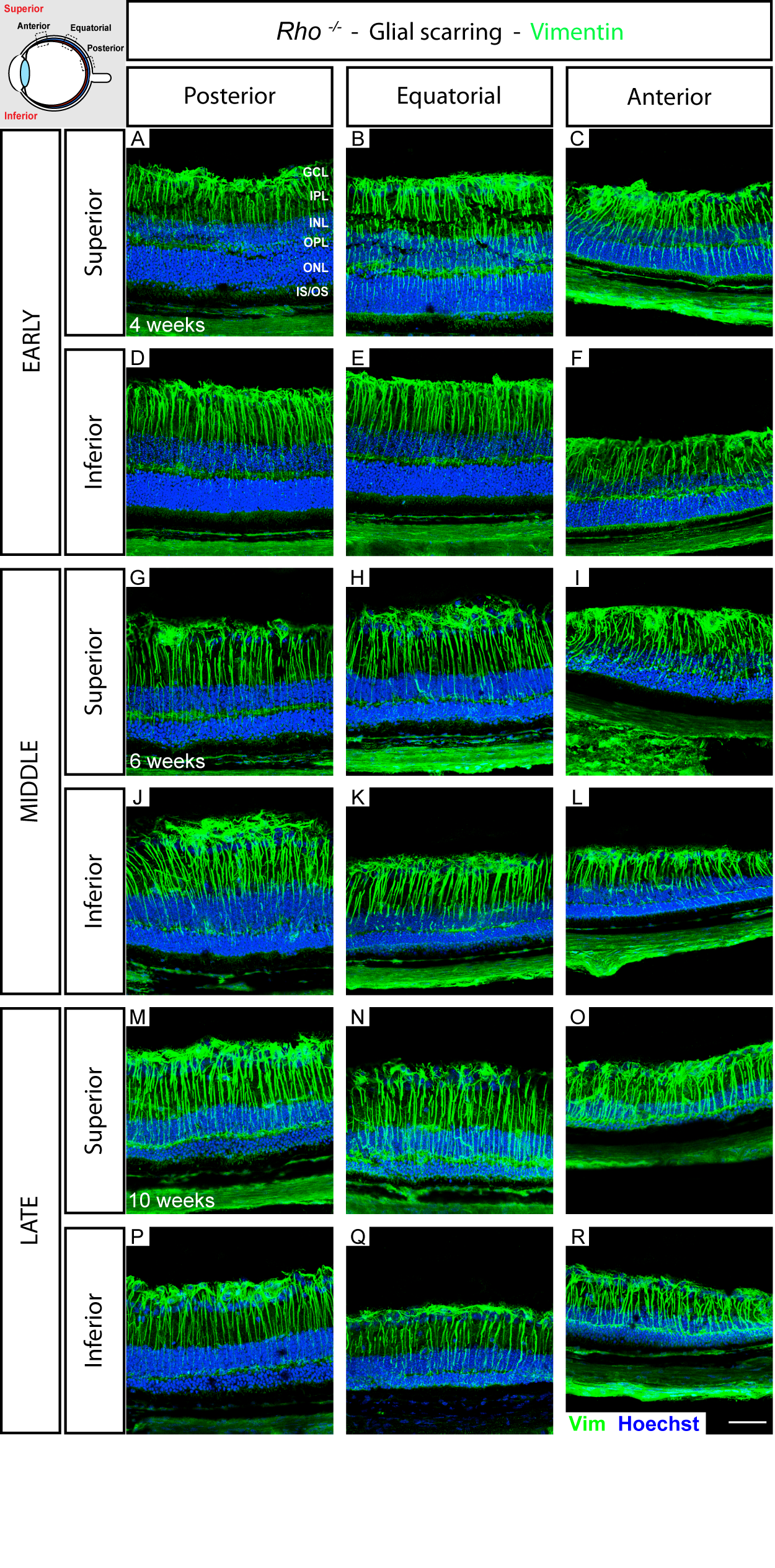

Supplement: S15 Fig — Cryosections were stained with glial cell marker vimentin (green) and counterstained with nuclei marker Hoechst 33342 (blue). Scale bar, 50 μm. (TIF) [file pone.0120415.s017.tif]

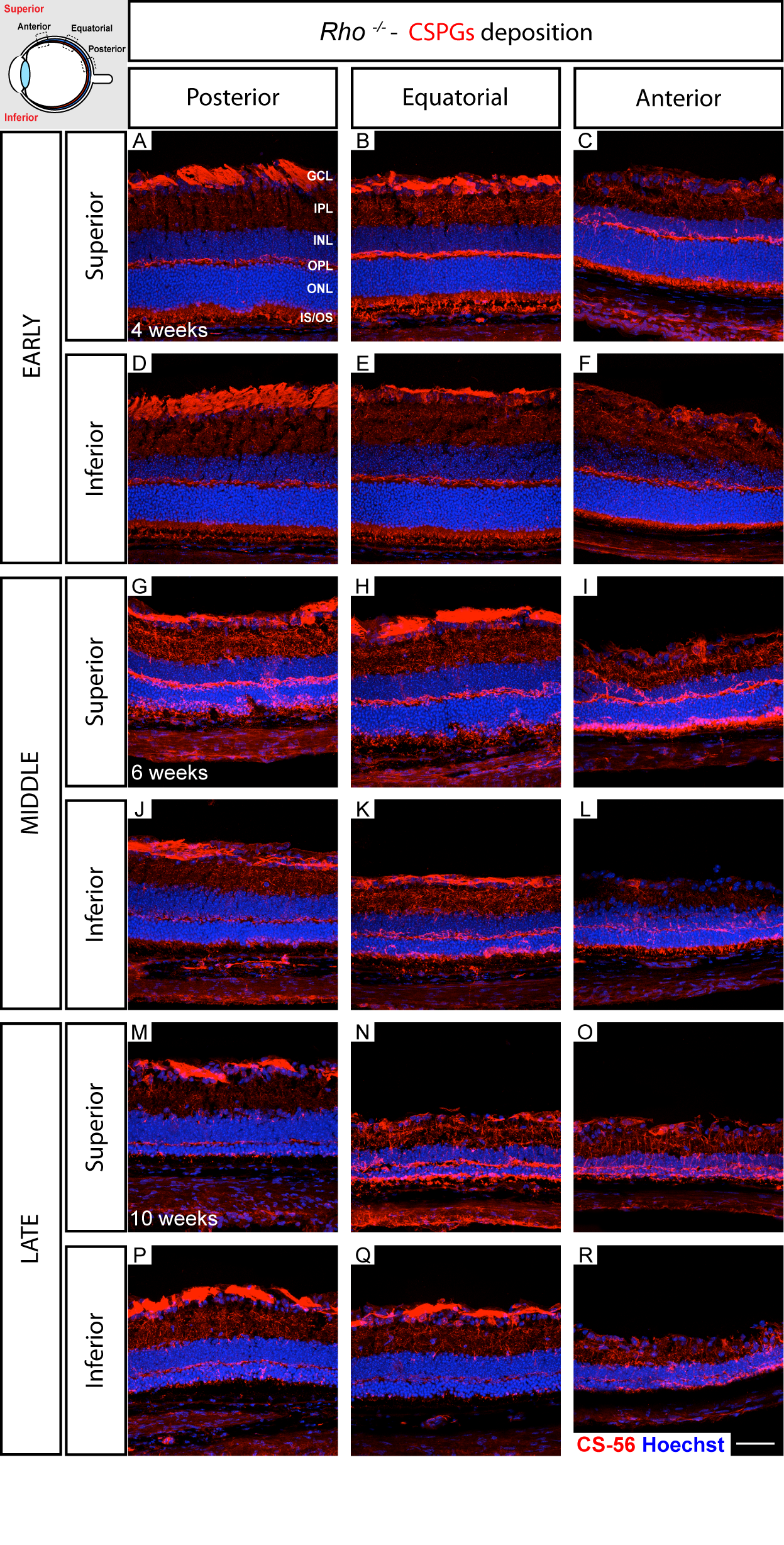

Supplement: S16 Fig — Cryosections were immunostained for CSPGs (CS-56, red) and counterstained with nuclei marker Hoechst 33342 (blue). Scale bar, 50 μm. (TIF) [file pone.0120415.s018.tif]

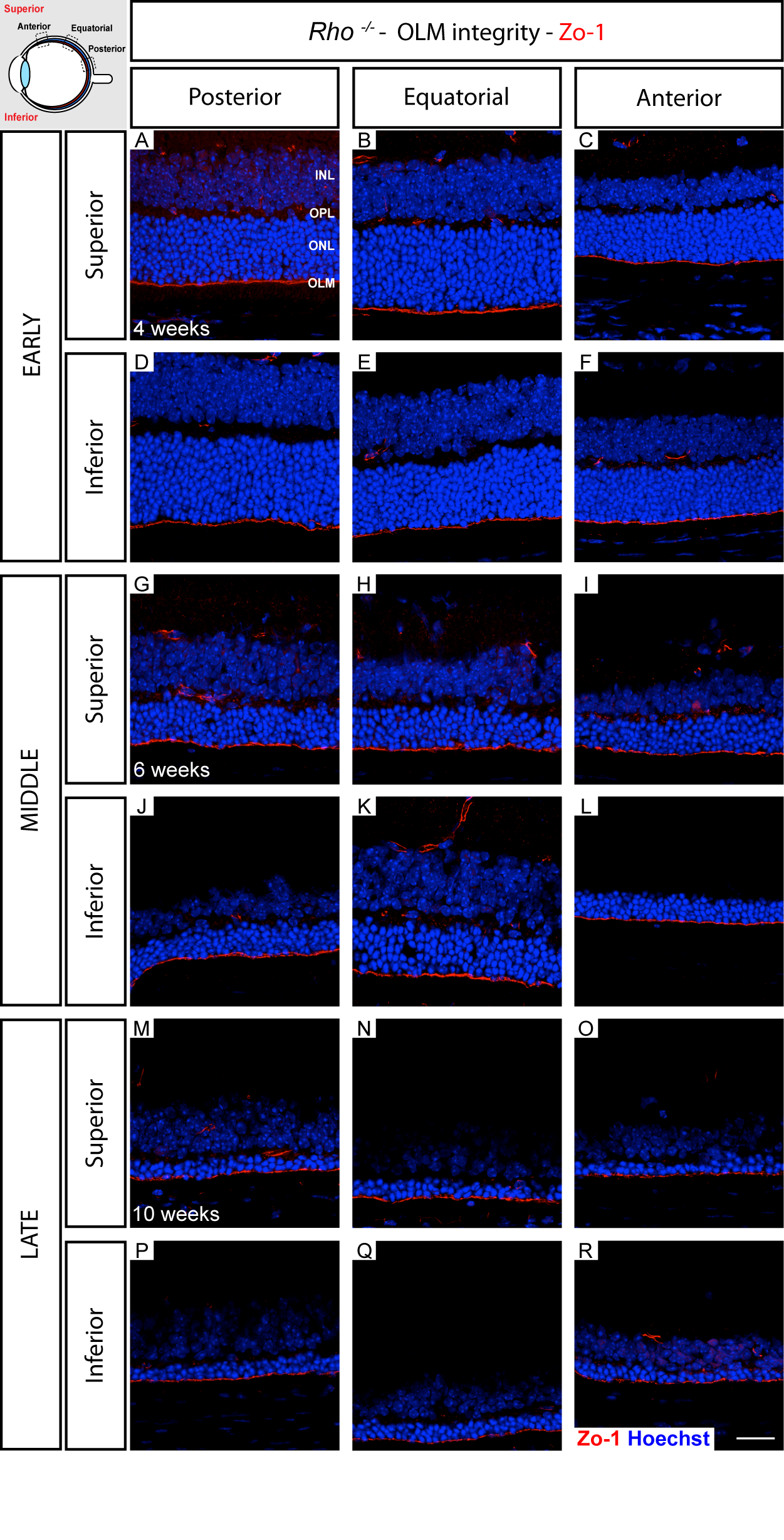

Supplement: S17 Fig — Cryosections were immunostained for Zo-1 (red) and counterstained with nuclei marker Hoechst 33342 (blue). Scale bar, 25 μm. (TIF) [file pone.0120415.s019.tif]

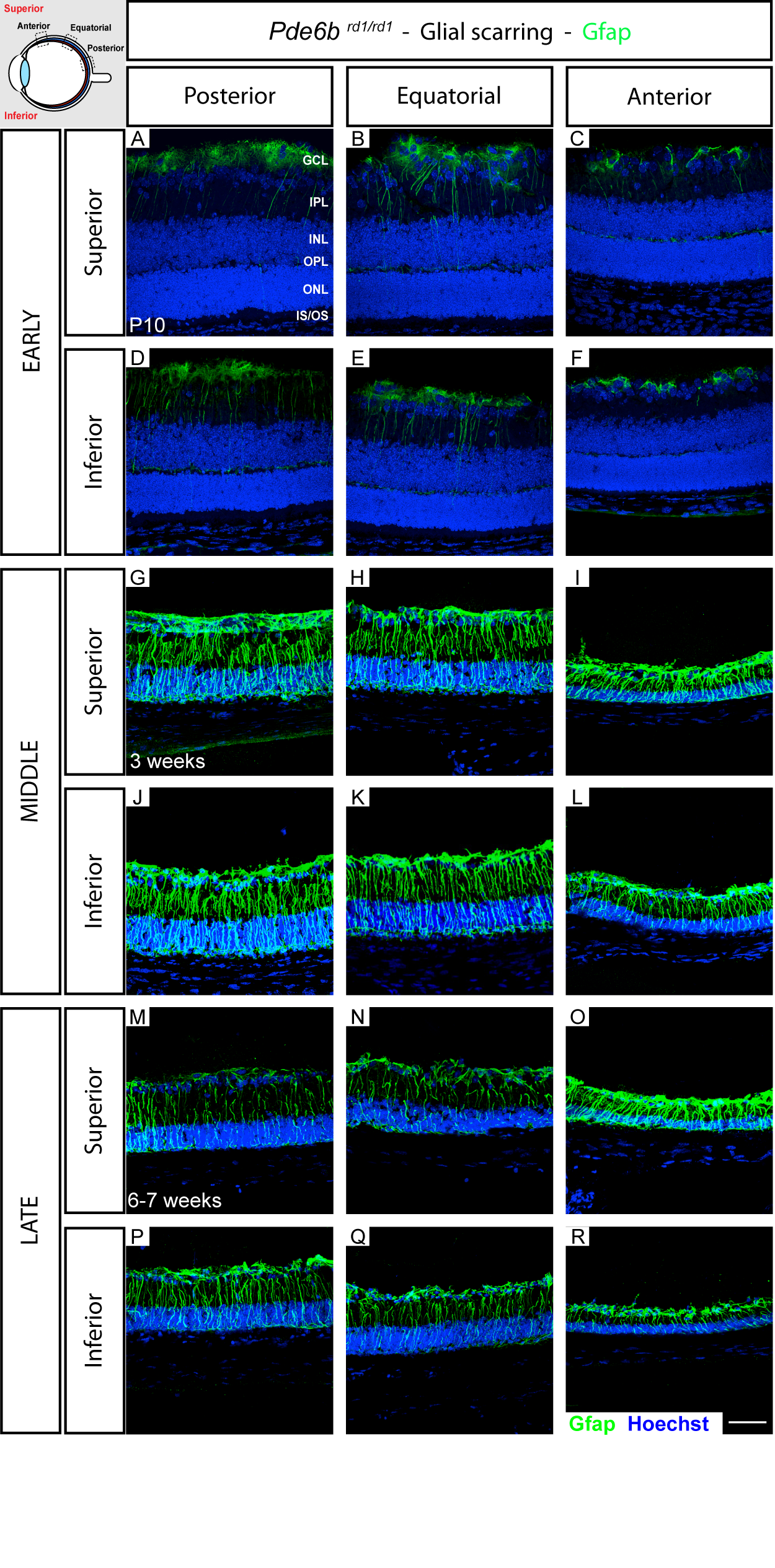

Supplement: S18 Fig — Cryosections were immunostained for glial cell marker Gfap (green) and counterstained with nuclei marker Hoechst 33342 (blue). Scale bar, 50 μm. (TIF) [file pone.0120415.s020.tif]

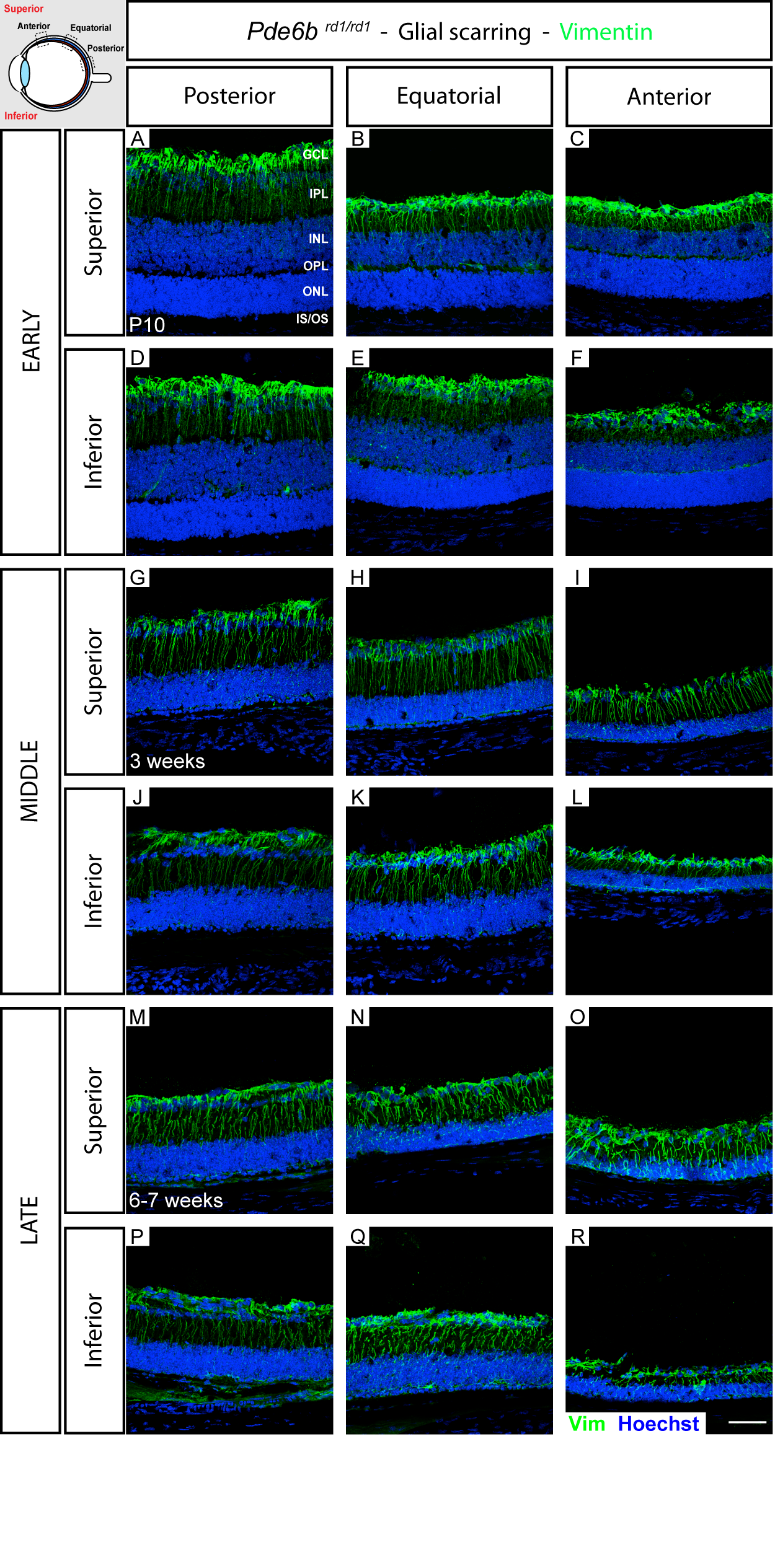

Supplement: S19 Fig — Cryosections were immunostained for glial cell marker vimentin (green) and counterstained with nuclei marker Hoechst 33342 (blue). Scale bar, 50 μm. (TIF) [file pone.0120415.s021.tif]

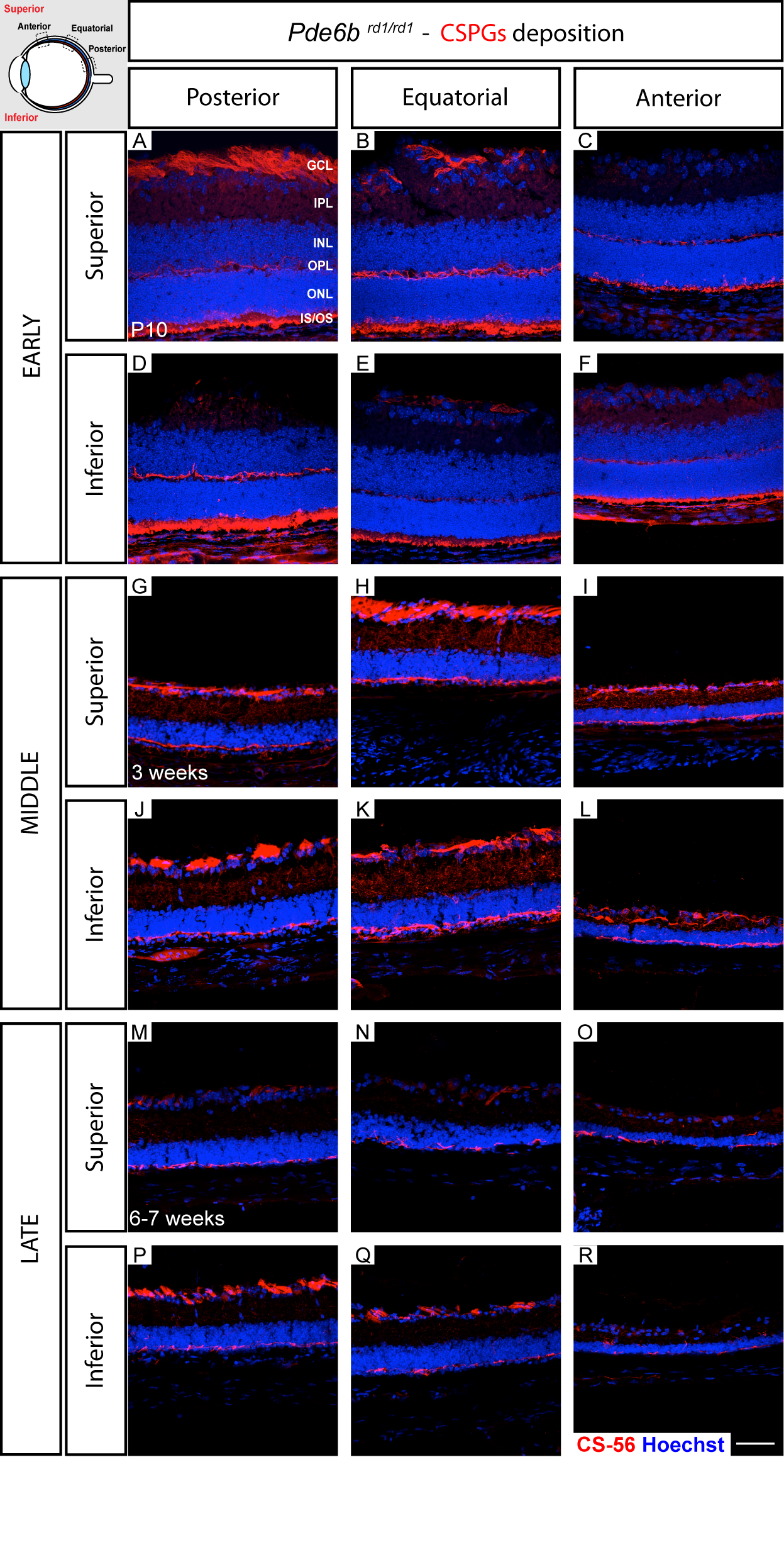

Supplement: S20 Fig — Cryosections were immunostained for CSPGs (CS-56, red) and nuclei marker (blue). Scale bar, 50 μm. (TIF) [file pone.0120415.s022.tif]

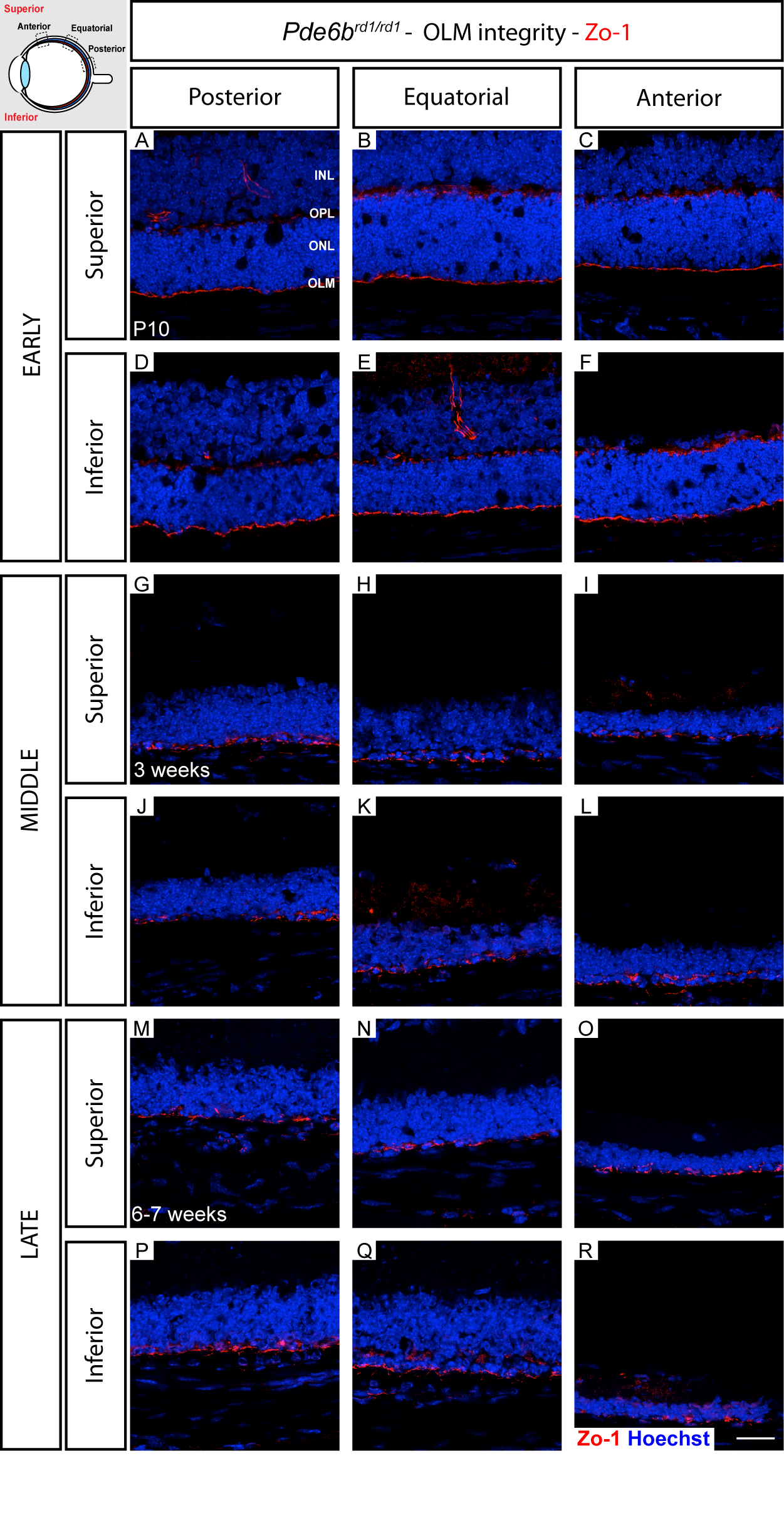

Supplement: S21 Fig — Cryosections were immunostained for Zo-1 (red) and counterstained with nuclei marker Hoechst 33342 (blue). Scale bar, 25 μm. (TIF) [file pone.0120415.s023.tif]

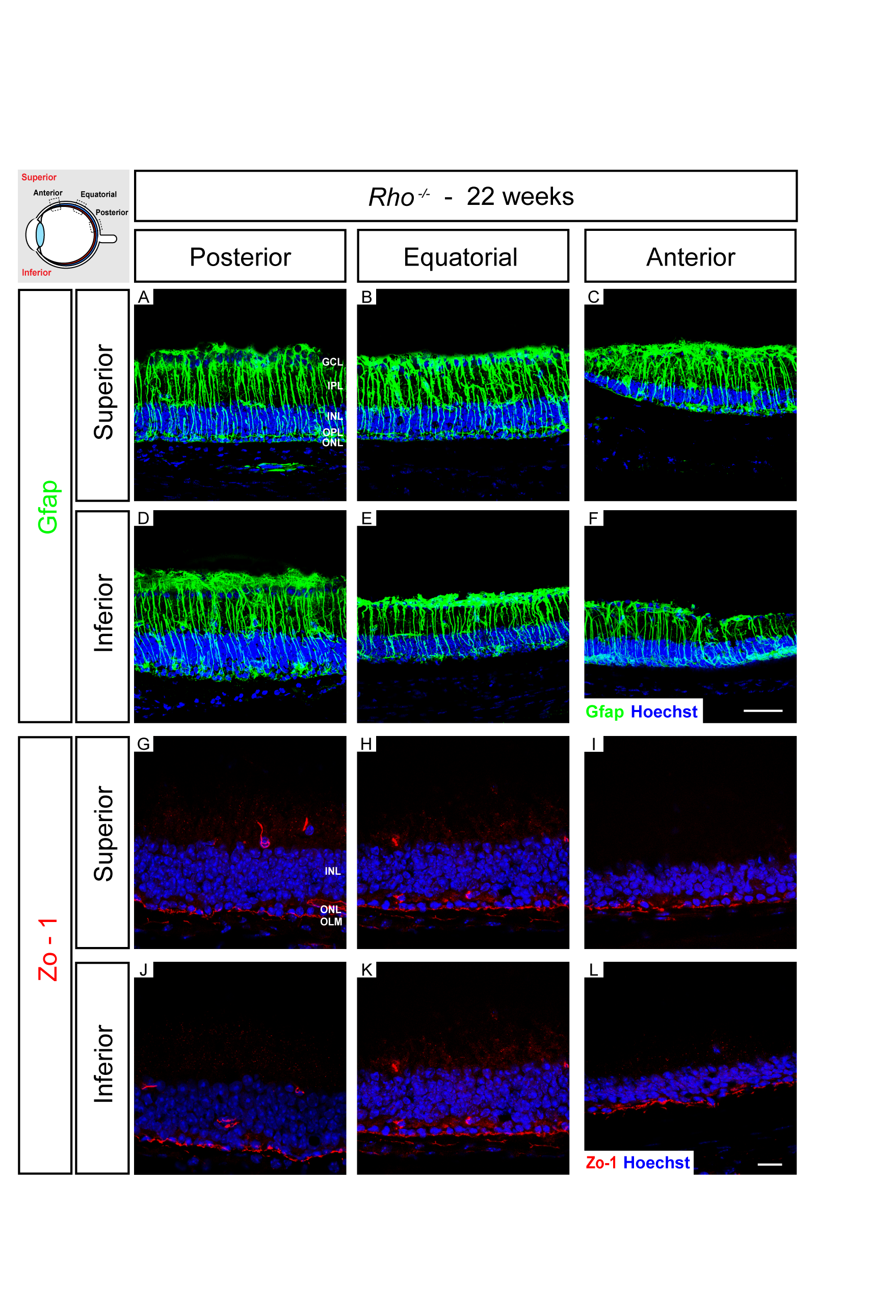

Supplement: S22 Fig — (A-F) At this age, high levels of Gfap were present throughout the Müller glia processes. At the outer edge of the retina, Gfap+ve processes were seen surrounding the remaining photoreceptor nuclei. (G-L) At this stage of degeneration, most of the photoreceptors are lost but the OLM remains largely intact. Cryosections were immunostained for glial cell marker Gfap (green) or Zo-1 (red) and co-stained with nuclei marker Hoechst 33342 (blue). Scale bar, 50 μm. (TIF) [file pone.0120415.s024.tif]

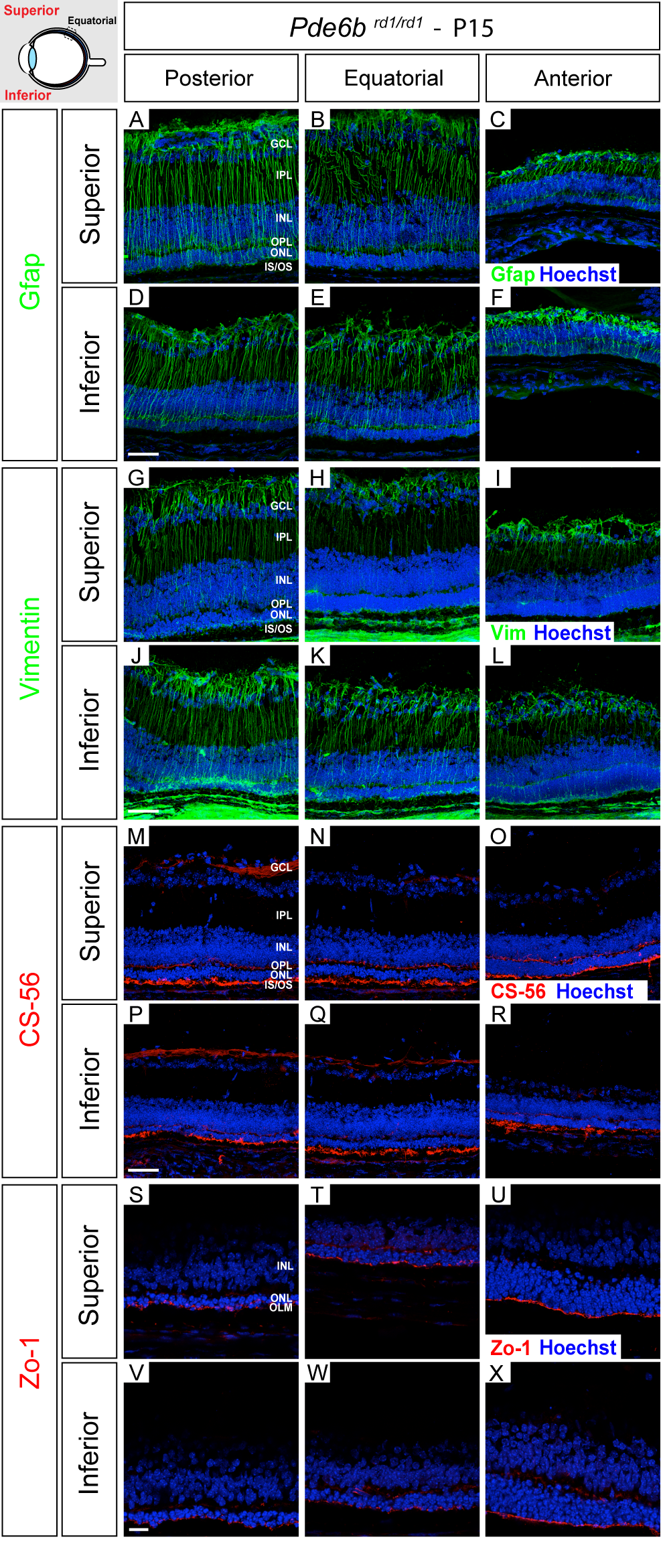

Supplement: S23 Fig — (A-F) In comparison to P10 retina, there was a marked upregulation in Gfap protein with glial processes extending towards the ONL. (G-L) Similarly, more vimentin+ve processes were observed extending towards the ONL. (M-S) At this stage, CSPGs were abundant in the subretinal space and the outer plexiform layer, but were reduced in the subretinal space in comparison to P10. (T-Y) In comparison to P10 animals, the OLM appeared more disrupted especially at the posterior regions. Cryosections were immunostained for glial cell marker Gfap (red) or vimentin (green) or CSPGs (red) and co-stained with nuclei marker Hoechst 33342 (blue). Scale bar, 50 μm. (TIF) [file pone.0120415.s025.tif]
